# A GIS-based on application of Monte Carlo and multi-criteria decision-making approach for site suitability analysis of solar-hydrogen production: Case of Cameroon

**DOI:** 10.1016/j.heliyon.2024.e41541

**Published:** 2024-12-27

**Authors:** Fotsing Metegam Isabelle Flora

**Affiliations:** a Environmental Energy Technologies Laboratory (EETL), Department of Physics, University of Yaounde I, P.O Box 812 Yaounde, Cameroon; b Department of Energetic, Environment and Thermal Engineering, UR-ISIE, University Institute of Technology Fotso Victor, University of Dschang, P.O Box 134, Bandjoun, Cameroon

**Keywords:** Solar energy hydrogen production, Monte Carlo, Geographical information system (GIS), Multi-criteria decision making (MCDM), Cameroon

## Abstract

This article analyzes and compares three methodologies for identifying suitable regions for solar hydrogen production using photovoltaic panels: AHP (Analytic Hierarchy Process), FAHP (Fuzzy Analytic Hierarchy Process), and MC-FAHP (Monte Carlo FAHP), integrated with GIS (Geographic Information Systems). The study employs ten criteria across technical (Global Horizontal Irradiance, temperature, slope, elevation, orientation), economic (distance from transportation and electrical networks), and social (population density, proximity to residential areas) factors. Environmental and exclusion criteria define restrictive zones. The analysis reveals that while all three methods agree on areas of low suitability, they diverge in their classification of "Suitable," "Highly Suitable," and "Most Suitable" regions. FAHP identifies 229.573 km^2^ as "Highly Suitable," compared to AHP's 222.048 km^2^ and MC-FAHP's 230.299 km^2^ for "Suitable" areas. Despite these differences, the energy potential is consistent across methods, totaling around 79,000 TWh/year, with MC-FAHP estimating the highest hydrogen production potential at 1.51 billion tons/year. The study concludes that fuzzy-based methods (FAHP and MC-FAHP) better handle uncertainties than traditional AHP. The MC-FAHP method, in particular, performs well in managing stochastic variability and yielding more reliable results. The findings are validated through a case study in Guider and Maroua, highlighting the importance of socio-economic and environmental criteria in decision-making. A sensitivity analysis reveals that economic and social criteria significantly influence land suitability, underscoring the importance of criteria selection in decision-making.

## Highlights

• Using solar energy in Cameroon for green hydrogen and electricity production.
• Using MCDM and Monte Carlo methods with GIS to improve decision-making in placing H2PV solar power plants.
• Introducing the MC-FAHP methodology in Cameroon to address local energy planning challenges.
• Diminishing Cameroon's dependence on imported petroleum by producing green hydrogen.

## 1 Introduction

Cameroon's abundant sunshine presents significant opportunities for the development of solar power plants aimed at producing green electricity and hydrogen. Solar energy is an eco-friendly resource that effectively mitigates greenhouse gas emissions, which are the primary drivers of climate change. Unlike fossil fuels, solar energy does not emit large quantities of carbon dioxide or other toxic pollutants, thereby helping to alleviate the adverse effects of global warming [1]. However, current energy storage technologies lack the versatility required for widespread applications across various sectors, as they are often designed for specific purposes. Additionally, renewable energy sources, such as solar, cannot guarantee the reliability of fossil fuels in future low-emission energy systems without an efficient energy storage infrastructure. Therefore, the development of effective and flexible technologies is essential to optimize renewable energy sources and decrease fossil fuel consumption in the energy sector [2]. Hydrogen plays a crucial role in this transition due to its capability to store excess solar energy, contributing to global decarbonization efforts and ensuring long-term sustainability [1]. This capability positions hydrogen for large-scale solar energy applications in Cameroon, particularly for energy storage and export. The potential applications of green hydrogen are diverse, including electricity generation, heating, transportation, and various industrial uses. Many countries are actively cultivating the green hydrogen industry to reduce CO2 emissions, create jobs, and establish export markets [3].

Despite its commitment to achieving the Sustainable Development Goals (SDGs) by 2030—specifically SDG 7, which aims to ensure universal access to reliable, sustainable, and modern energy services at an affordable price—Cameroon faces numerous challenges. The country has identified energy as a critical component of its development and emergence policy. According to the Global Energy Architecture Performance Index, Cameroon has an electrification rate of 54 % overall, which drops significantly in rural areas, where only 3,757 of the 14,207 localities (approximately 26 %) are connected to electricity. Nevertheless, Cameroon possesses substantial potential for energy generation from diverse sources [4].

Ministry of Water and Energy (MINEE) reports that Cameroon has an installed capacity of 1.3 GW, primarily from large hydroelectric and natural gas plants. In 2014, total electricity production was estimated at 7,688.45 GWh, with the following sources: hydroelectricity (57.56 %), public thermal (21.6 %), thermal self-production (20.79 %), and renewable energies (0.1 %) [5]. By 2019, the primary energy source distribution in Cameroon was as follows: biomass (53 %), hydroelectricity (3.5 %), and fossil fuels (43.2 %), with oil accounting for 28.3 % and natural gas for 14.8 %. Notably, Cameroon exports 90.5 % of its crude oil and 70 % of its natural gas, while two-thirds of the petroleum products consumed domestically are imported [6].Harnessing renewable energy, particularly solar—whose potential is estimated at 2,327 TWh/day—represents a strategic pathway for Cameroon to achieve its goal of integrating 25 % renewables into the electricity mix by 2035, contributing to a 32 % reduction in greenhouse gas emissions [6]. Given the current climate challenges, renewable energy emerges as a viable and sustainable solution to address Cameroon's energy deficit. By leveraging its abundant solar resources, Cameroon has the opportunity to develop a robust green hydrogen industry, reducing its dependence on oil imports and enhancing the reliability of its energy system to accommodate more renewable sources. However, the country has yet to establish a comprehensive green hydrogen strategy that outlines development plans and sets targets to stimulate this industry and attract investment. Formulating policies and objectives for the growth of this innovative sector in Cameroon necessitates a thorough assessment of green hydrogen potential, which will inform the development of a roadmap to encourage investor interest. Identifying suitable sites for smart hydrogen solar power plants in Cameroon will involve evaluating technical, economic, social, and environmental factors.

While several studies have examined hydrogen production using solar energy, much of the existing research has focused on countries with more developed renewable energy infrastructures, such as Turkey, Algeria, Mexico, and Morocco. These studies primarily aim to evaluate technical and economic factors for hydrogen production, including optimal site selection, system configurations, and electrolysis processes. For example, Hasan and Genç [7] analyzed the cost and efficiency of hydrogen production from solar and wind energy, while Karipoğlu et al. [8] focused on GIS-based site selection for hydrogen charging stations in Turkey. Other works, such as those by Messaoudi et al. [9] and Rahmouni et al. [10], explored land suitability for hydrogen production in Algeria, and Juárez-Casildo et al. [11] assessed the hydrogen production potential across Mexico. Amrani et al. [12] evaluated Morocco's hydrogen production potential, finding solar photovoltaic (PV) to be more advantageous than concentrated solar power (CSP), with 20.3 % of land suitable for PV hydrogen production, compared to 7.4 % for CSP. A 1 MW H2PV plant could generate 48.5 tonnes of hydrogen annually at a cost of $4.972/kg. Amjad et al. [13] estimated Pakistan's potential to produce 7 million tonnes of hydrogen per year from solar PV. Nematollahi et al. [14] analyzed wind and solar data in Iran's Sistan and Baluchistan province, suggesting small wind turbines could produce 39.7 tonnes of hydrogen annually, with lower electricity costs. G. Zhang et al. [15] introduced a hybrid method to determine the optimal location and size for an off-grid solar/hydrogen system in Iran, considering social, economic, and environmental criteria. Taoufik and Fekri [16] studied solar hydrogen potential in Morocco's Souss-Massa region using AHP and GIS, providing valuable insights through sensitivity analysis. Kalbasi et al. [17] conducted a techno-economic study in Iran, finding the southern regions most favorable for hydrogen production from solar energy. These studies, though important, predominantly apply to contexts with well-established energy systems or aim to optimize existing technologies for specific local conditions.

In Cameroon, several studies have also been conducted on renewable hydrogen production. Wankouo Ngouleu et al. [18] performed a comparative evaluation of meta-heuristic optimization methods to design hybrid photovoltaic/wind turbines/fuel cell systems and photovoltaic/wind turbines/hydrogen battery systems to meet three current load demands in Kousseri, Cameroon. They concluded that the autonomous photovoltaic/wind/battery hybrid system is the most reliable and cost-effective solution for satisfying electrical load demands in the region. Sapnken et al. [19] assessed the potential of green hydrogen (H2) as an alternative fuel for the Cameroonian road transport system, estimating that transitioning to hydrogen fuel by 2035 would require between 1.75 and 2.5 million tonnes of hydrogen annually. Koholé et al. [20] examined wind potential for electricity and hydrogen production in several cities in the Far North region of Cameroon, utilizing wind speed data recorded at various heights.

Identifying suitable locations for renewable energy installations is a critical step for advancing solar energy sustainably. However, this task is complicated by the need to balance numerous conflicting criteria. The Multi-Criteria Decision-Making (MCDM) method has emerged as an effective tool for evaluating and ranking alternatives for renewable energy projects. Numerous studies have applied MCDM methods to identify optimal sites for solar power installations. For example, Hosseini Dehshiri and Firoozabadi [21] developed a four-step framework combining GIS and fuzzy MCDM to identify ideal areas for solar plants in Iran's dust-prone regions, highlighting Yazd province's potential for electricity and hydrogen production. Nagababu et al. [22] proposed a GIS-MCDM algorithm, using the Fuzzy Analytical Hierarchy Process (FAHP) and the Technique for Order Preference by Similarity to Ideal Solution (TOPSIS) to identify suitable wind farm sites, considering technological, economic, social, and environmental factors. Minaei et al. [23] applied a GIS-based approach and the Best Worst Method (BWM) to assess areas suitable for solar photovoltaic systems, particularly for rural energy needs.

Several criteria have been used to pinpoint optimal locations for solar power plants. Akinci and Özalp [24] considered factors such as solar radiation, slope, proximity to roads and transmission lines, and land use to identify suitable sites in Turkey. Hooshangi et al. [25] introduced a novel approach using GIS-based Fermatean Fuzzy (FF) TOPSIS, prioritizing sites by considering economic, geographic, climatic, infrastructural, and demographic criteria. Türk et al. [26] employed ten criteria including slope, aspect, and land use, combining intuitionistic fuzzy sets with GIS for solar plant site selection in Erzurum Province, Turkey. Demir et al. [27] proposed an innovative method using AHP and GIS for large-scale PV plant site selection, introducing the Optimality Based Site Growth (OBSG) technique. Similarly, Rekik and El Alimi [29] assessed potential sites for wind and solar plants in Tunisia using GIS and MCDM methods. Saeidi et al. [30] utilized TOPSIS and fuzzy-Boolean logic in GIS for solar PV site identification in Tehran Province. Nitin Liladhar et al. [31] use a GIS-based Multi-Influencing Factor (MIF) approach to identify optimal locations for solar PV farms in Nashik, India. In another study [32], they apply the same MIF technique to determine ideal sites for future urban settlements in Nashik. The fuzzy method has been successfully applied by various authors to identify suitable sites for renewable energy plants across different countries [[33], [34], [35], [36], [37], [38], [39]].

Traditional GIS-MCDA methods are widely used but often suffer from high randomness and uncertainties, leading to unreliable results [40]. Analytical Hierarchy Process (AHP) is commonly used to determine the weights of criteria in these methods but is criticized for not fully addressing the uncertainties in pairwise comparisons. To improve decision-making, advanced techniques like the Enhanced Analytical Hierarchy Process (EAHP), Fuzzy AHP (FAHP), and Entropy Weighting Methods have been proposed to handle the uncertainties in weight assignment. Despite these improvements, uncertainty in weight determination remains a significant challenge. Monte Carlo simulations provide an effective approach for managing uncertainties by incorporating random fluctuations in environmental parameters. However, few studies have applied Monte Carlo simulations alongside AHP and GIS for site selection. Notable examples include Wang et al. [41], who combined Monte Carlo simulation, AHP, and GIS to assess site suitability for oyster reef restoration in China. Butschek et al. [42] used Monte Carlo simulations for offshore wind energy site selection in Ireland, while Dahri & Abida [43] applied AHP and Monte Carlo simulations to flood vulnerability mapping in Tunisia. Similarly, Adam et al. [44] used GIS and Monte Carlo simulations with AHP for Managed Aquifer Recharge (MAR) site evaluation, and Feizizadeh & Blaschke [45] combined MCDA-GIS methodologies with Monte Carlo simulations to map landslide susceptibility in Iran, emphasizing the reliability of MCDA results in environmental studies.

This study sets itself apart from previous research by addressing the unique energy challenges and opportunities in Cameroon, a country with abundant solar resources but limited infrastructure for renewable energy storage. While much of the existing literature focuses on countries with established renewable energy systems, such as Morocco, Algeria, and Turkey, this research tackles the specific constraints faced by Cameroon, including the absence of a comprehensive green hydrogen strategy.

The primary objective of this study is to identify optimal sites for hybrid photovoltaic (H2PV) solar power plants for green hydrogen production, using advanced multi-criteria decision-making (MCDM) techniques—namely the Analytic Hierarchy Process (AHP), Fuzzy AHP (FAHP), and Monte Carlo Fuzzy AHP (MC-FAHP)—coupled with Geographic Information System (GIS) mapping. This methodological integration allows for a more accurate and robust evaluation of potential sites, incorporating uncertainties and multiple influencing factors, such as solar irradiance, infrastructure access, environmental constraints, and socio-economic considerations. This study represents a significant innovation in applying a combined MC-FAHP methodology for site selection in the context of Cameroon, a country where the green hydrogen sector remains in its infancy. Unlike previous studies that may focus on specific renewable energy sources or applications, the current research introduces a comprehensive, multi-method approach to address the unique challenges Cameroon faces, such as limited energy access, a lack of renewable energy infrastructure, and the need for a sustainable hydrogen production strategy to diversify the energy mix.

Moreover, while much of the existing research on solar hydrogen production has been conducted in regions with relatively well-defined renewable energy policies and advanced technical systems, Cameroon's energy landscape presents a distinct challenge. The country has abundant solar resources but lacks the necessary energy storage infrastructure, particularly for green hydrogen. This study aims to fill this gap by integrating the potential of green hydrogen into Cameroon's broader energy strategy, offering a pioneering framework to assess and select optimal sites for large-scale solar-powered hydrogen production. By addressing key factors such as solar irradiance, infrastructure access, and environmental constraints, the study provides a novel pathway for sustainable energy development in Cameroon, with implications for reducing fossil fuel dependency and promoting green energy innovation in Africa.

Following a thorough review of existing literature, Table 1 outlines the criteria and methodologies commonly used in global renewable energy site identification, highlighting predominant factors such as solar irradiation, proximity to electrical grids, temperature, land slope, distance from residential areas, and proximity to water bodies. Ten factors—including Global Horizontal Irradiance (GHI), temperature, slope, aspect, elevation, distances to power lines, residential areas, power plants, road networks, and population density—along with several restrictive criteria were meticulously selected based on literature reviews and expert consultations for this study.

In summary, while previous studies have explored various technical and geographical aspects of renewable hydrogen production, the current study distinguishes itself by focusing on Cameroon's specific needs, applying advanced methodologies to account for uncertainties in site selection, and offering a strategic blueprint for integrating solar power and hydrogen into a sustainable energy system.

The structure of this article is as follows: Section 1 provides a comprehensive introduction along with a thorough literature review. Section 2 presents the study area and details the data and methods employed (AHP, FAHP, MC-FAHP, GIS). Section 3 discusses the results and implications, followed by conclusions in Section 4.

## 2 Materials and methods

**Fig. 1:**
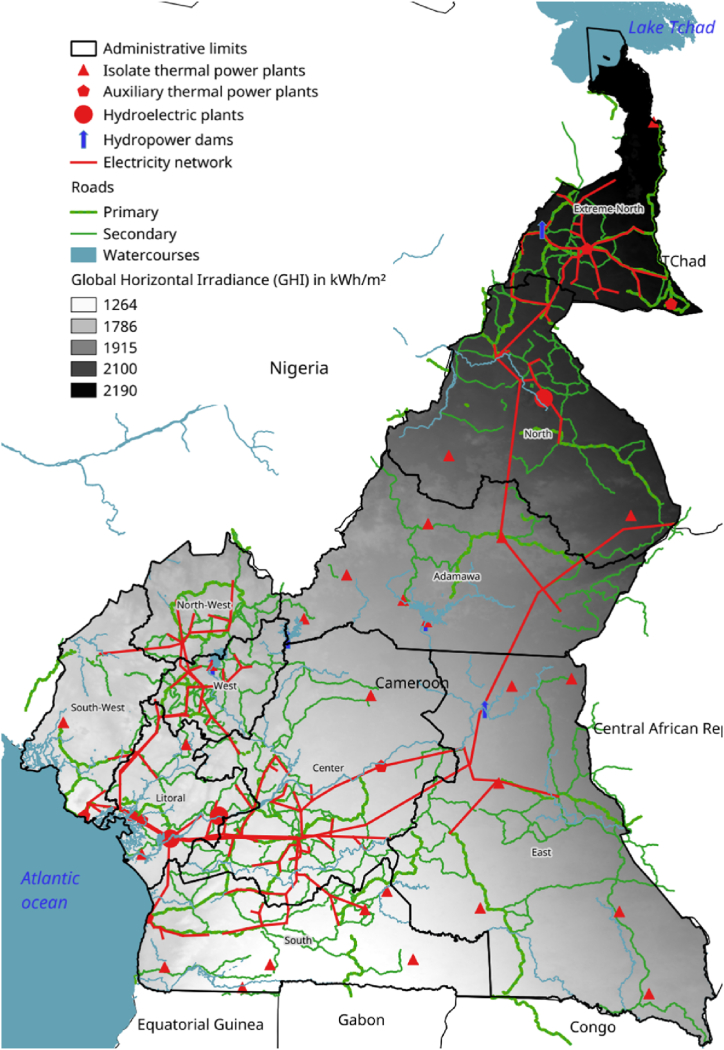
Energy map of Cameroon.

In this section, the study area of this research will be presented. This will be followed by an introduction to solar photovoltaic (PV) technology for hydrogen production. The study data and methodological framework will also be presented. The methodology used in this study will include three key steps: calculating the weight of different criteria using the AHP (Analytic Hierarchy Process), the FAHP (Fuzzy Analytic Hierarchy Process) and the MC-FAHP (Monte Carlo Fuzzy Analytic Hierarchy Process) for the selection of the sites most suitable for the construction of H2PV solar parks. Fig. 1 illustrates an energy map of Cameroon with Global Horizontal Irradiance (GHI) as a backdrop.

**Fig. 2:**
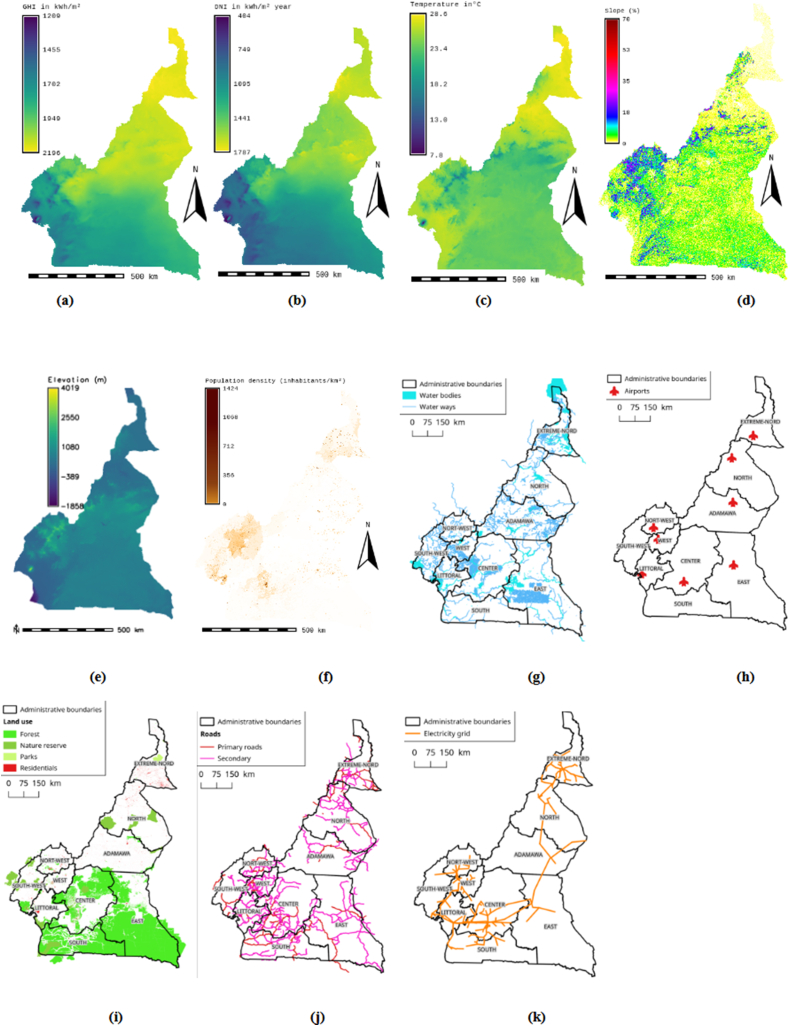
Study data.

### 2.1 Field of study

Cameroon is situated between latitudes 2° and 13° North, and longitudes 8° and 16° East. Geographically, it forms a right triangle, with its hypotenuse stretching over 1,500 km from Lake Chad to the Gulf of Guinea, and its base extending 800 km along the Atlantic Ocean, bordering the Central African Republic. Cameroon shares borders with several countries: to the north, it is bordered by Lake Chad; to the northeast, by the Republic of Chad; to the east, by the Central African Republic; to the south, by the Republic of Congo, Gabon, and Equatorial Guinea; and to the west, by Nigeria [54]. The country's landscape is highly diverse, with scattered highlands surrounded by narrow plains. In the far north, the Mandara Mountains reach an average altitude of 1,000 m, while the Adamawa Plateau, located near the center of the country, rises to 1,100 m. The western highlands feature plateaus at altitudes between 1,200 and 1,800 m, alongside a mountain range that extends from the Atlantic coast. Notable volcanic peaks in this region include Mount Cameroon, an active volcano and the highest point in the country at 4,070 m; Mount Manengouba (2,396 m); the Bamboutos Mountains (2,740 m); and Mount Oku (3,008 m). These highlands descend to the South Cameroonian plateau, which has an average elevation of 650–900 m [55]. Cameroon is divided into three major climatic zones. The equatorial zone, which experiences abundant rainfall (around 2,000 mm annually), has an average temperature of about 25 °C. The Sudanian zone, characterized by a long dry season (lasting five to six months), receives about 1,000 mm of rainfall per year and has an average temperature of 22 °C. The Sudano-Sahelian zone, located further north, endures an extended dry season with more arid conditions [5]. The energy sector plays a crucial role in Cameroon's development and its emergence policy. According to the Global Energy Architecture Performance Index, Cameroon's electrification rate stands at 54 % [56]. However, in rural areas, this rate is significantly lower, with only 3,757 of 14,207 localities being electrified, representing just 26 %, as per WSP-RAINBOW data from April 2016 [57]. Despite these challenges, Cameroon has considerable potential to produce energy from diverse sources. Data from the Ministry of Water and Energy (MINEE) shows that the country's installed energy capacity stands at 1.3 GW, primarily sourced from large hydroelectric and thermal power plants. In 2014, the country produced a total of 7,688.45 GWh of electricity, distributed as follows: 57.56 % from hydroelectricity, 21.6 % from public thermal plants, 20.79 % from self-produced thermal energy (both onshore and offshore), and just 0.06 % from renewable energy sources [6].

### 2.2 Solar PV technology for hydrogen production

Research into hydrogen production from photovoltaic (PV) solar energy presents promising prospects for more cost-effective and sustainable methods. Many researchers have focused on improving the competitiveness of this technology. For instance, Lubbe et al. (2022) [58],developed a system that produces green hydrogen using specialized solar panels. These panels extract moisture from the air and convert it into hydrogen without relying on rare metals or water, achieving an efficiency of approximately 15 %. In 2023, Lubbe et al. [59] further explored hydrogen production via modular solar generators, alongside centrally produced green hydrogen. They demonstrated that solar hydrogen generators are inherently modular and suitable for decentralized applications, scalable from kilowatts to gigawatts. The versatility of these generators offers several advantages, including reduced financial risk, faster innovation cycles, job creation, and lower CO2 emissions. Despite these benefits, green hydrogen remains more expensive than gray or blue hydrogen due to the high costs of renewable energy and water electrolyzer efficiency [60]. To address the cost challenge, Ahmad et al. (2023) [60] proposed the use of bifacial PV technology to boost green hydrogen production. This approach integrates bifacial PV systems with cool roof technology (high-albedo roofs) to maximize both energy and hydrogen output. Similarly, Zhang et al. [61] developed a solar cell capable of producing hydrogen and oxygen through water electrolysis, offering a more cost-effective solution compared to high-efficiency solar cells made from materials like amorphous silicon. Their system converts 5 % of light energy into hydrogen. Ardo et al. [62] created a device that focuses solar rays using a parabolic mirror to generate large amounts of hydrogen. This system achieves a solar-to-hydrogen conversion efficiency of over 17 % through an advanced heat exchange mechanism. Additionally, Jacques Hass [63] explored hydrogen generation using sunlight and water through processes mimicking natural photosynthesis. Although the efficiency of this method is still under development, it presents a simple and economical way to convert solar energy into hydrogen, which can be stored for later use in fuel cells or converted into methane.

Since the early 2000s, the photovoltaic industry has experienced rapid growth, driven by advances such as improved cell efficiency, lower production costs, and the development of new technologies like thin-film and perovskite solar cells [64]. Large-scale PV installations have become increasingly competitive with other energy sources and are being integrated more extensively into electrical grids. PVsystems are now widely recognized as a sustainable and viable energy source, supporting the global transition toward a greener economy. Various PV systems are employed for large-scale electricity generation. Ground-mounted solar power plants, for example, cover vast areas and can be installed in deserts, agricultural lands, or other underdeveloped regions. Tracker solar power plants use structures that enable the solar panels to follow the sun's movement throughout the day, optimizing sunlight exposure and boosting electricity production. Floating solar power plants, which consist of panels mounted on floating structures on bodies of water such as lakes or reservoirs, represent another innovative solution. Rooftop solar power plants utilize available roof space to generate electricity, while building-integrated PV systems replace conventional roofing materials with solar panels [65]. However, large-scale solar electricity production faces challenges, particularly due to the intermittent and uneven distribution of solar irradiation [66]. As a result, research into identifying optimal sites for H2PV solar power plants is essential. Hydrogen produced at these locations can be used as an energy storage medium, helping to stabilize the electricity grid and ensure a more reliable energy supply.

### 2.3 Site selection criteria evaluation and constraint map

Advancements in spatial analysis techniques have significantly improved their reliability, efficiency, and cost-effectiveness as tools for evaluating the potential of renewable energy. These techniques facilitate the assessment of various spatial factors, such as resource availability, topography, land use, infrastructure, and environmental considerations, thus enhancing the precision of renewable energy evaluations and aiding informed decision-making in project planning. This method ensures a comprehensive evaluation of the feasibility of hybrid installations, optimizing energy production while minimizing associated risks and costs. The study examines variables like GHI solar radiation, elevation, topographical slope, population density, land use/cover, average air temperature, proximity to transmission lines and substations, distance to water bodies, and residential areas.

### 2.4 Boolean logic method

The research utilizes Boolean logic to develop a suitability model that categorizes areas as suitable (assigned a value of "1″) or unsuitable (assigned a value of "0″). Based on Boolean logic, this model is exact and facilitates delineating a constraints layer that embodies restrictive criteria. The constraints layer is instrumental in excluding unsuitable locations from the final suitability map. Table 10 presents the restrictive criteria used to generate the constraints map for H2PV solar power plant sites. The final constraints map is illustrated in Fig. 5.

### 2.5 Fuzzy sets method

Fuzzy logic is employed in multi-criteria analysis to manage uncertainty and imprecision, enabling expert systems to convey complex information in an interpretable format. This approach is based on the fuzzy set theory introduced by Zadeh [87,88]. A fuzzy subset A of a set E is defined by a membership function that maps each element of E to a value between 0 and 1, representing the degree to which an element belongs to subset A. Unlike traditional sets, where an element either belongs to a subset or not (as indicated by 0 or 1 in a characteristic function), fuzzy logic assigns intermediate values, reflecting partial membership. Fuzzy logic is particularly effective in addressing the uncertainties and imperfections inherent in geographic data, helping to minimize subjectivity in assessments. By combining fuzzy logic with multi-criteria analysis techniques within a GIS environment, decision-making processes are enhanced, producing more realistic and reliable outcomes. Common membership functions used in identifying suitable locations for renewable energy include linear, triangular, trapezoidal, Gaussian, and sigmoid functions [24,38,76,89]. In fuzzy decision-making models, the selection of membership functions is essential for effectively representing uncertainty and imprecision in data. This study employs linear, triangular, and trapezoidal membership functions due to their simplicity, computational efficiency, and suitability for handling various real-world fuzzy set applications. While Gaussian membership functions are better suited for scenarios with well-defined central values and less clear extremes, they are more complex, less intuitive, and harder to interpret in practical decision-making contexts. Consequently, simpler functions like linear, triangular and trapezoidal were preferred in this research. The trade-off between simplicity and precision leans toward the latter, particularly when interpretability and computational efficiency are paramount. The properties and characteristics of these fuzzy membership functions, as outlined in Table 4, are crucial in defining the criteria for site selection, ensuring a robust and flexible evaluation process.

### 2.6 AHP method

In the 1970s, Thomas Saaty introduced the Analytic Hierarchy Process (AHP), a decision-making framework designed to simplify complex decisions by breaking them down into smaller, more manageable components [90]. AHP is particularly suited for multi-criteria decision-making problems, where multiple solutions must satisfy a set of defined criteria. The steps involved in the AHP process are illustrated in Step 2 of Fig. 4. AHP has been successfully employed by various researchers to identify optimal sites for solar power plants dedicated to green hydrogen production worldwide [2,9,12,16,40]. Table 5 presents a fundamental numerical scale ranging from 1 to 9, which enables the assessment of both the quantitative and qualitative performance of priorities. In this study, ten solar energy experts from academia and industry were invited to complete a pairwise comparison matrix, evaluating criteria and sub-criteria. They assigned values from 1 to 9, indicating the relative importance of each parameter in comparison to others, as shown in Table 5. XLSTAT software was used to collect and process the input data matrix. The results of the AHP analysis, including the weights assigned to the main criteria and sub-criteria, were extracted, along with the consistency ratio (CR) and consistency index (CI). The AHP results are presented in Table 8, which includes the CI value of 0.145 and a CR of 9.72 %. Since the CR is below the threshold of 10 %, the consistency of the comparisons is considered acceptable, validating the reliability of our study's findings.

### 2.7 Fuzzy Analytic Hierarchy Process (FAHP) method

Fuzzy-AHP is a multi-criteria decision-making method that integrates the traditional Analytic Hierarchy Process (AHP), developed by Saaty in 1977 [90], with fuzzy set theory. Initially introduced by van Laarhoven in 1983 [88] and further refined by Chang in 1996 [91], this approach enables decision-makers to assess the relative importance of various criteria and alternatives in scenarios involving uncertainty or imprecision. Unlike AHP, which relies on precise numerical values, Fuzzy-AHP utilizes fuzzy numbers that reflect linguistic expressions, accommodating ambiguous or uncertain judgments. This method assigns degrees of membership to values, indicating the extent to which they belong to a particular expression. Fuzzy-AHP is especially useful in contexts where evaluations are subjective or data is imprecise, allowing for a more flexible and nuanced analysis. The Saaty scale is still employed in decision-making but is adapted to fuzzy logic. Table 6 provides Saaty's scale as applied in Fuzzy-AHP. Fuzzy-AHP is widely recognized for its effectiveness in determining the weights of various criteria, which are then used to calculate different suitability indices. It has been applied in numerous studies to optimize site selection for renewable energy plants worldwide [21,23,24,25,26,30,35,36,37,39,76,89,92,93]. The process steps for Fuzzy-AHP are outlined in Step 3 of Fig. 4.

Table 8 presents the results of the Fuzzy-AHP analysis, including the consistency index (CI) of 0.099 and a consistency ratio (CR) of 6.67 %. Since the CR is below the 10 % threshold, the results of this study are considered valid and reliable.

### 2.8 Monte Carlo simulation

The installation of solar power plants for electricity and hydrogen production is fraught with uncertainties related to their inherent characteristics. These projects, often emerging from a sector with limited operational experience, face uncertainties from the early design and development phases [53]. Therefore, assessing the uncertainties associated with site selection indicators is vital for informed decision-making, as it facilitates a comprehensive analysis that supports sound conclusions. In this context, the Monte Carlo method can be employed to evaluate the risks and uncertainties linked to the project, considering variations in climatic, orographic, economic, and socio-environmental factors.

Probability theory has long been utilized to characterize random variables and uncertain phenomena. Monte Carlo simulation, which relies on statistical data, is regarded as a realistic approach due to its incorporation of random sampling from a probability distribution. This method is particularly useful for managing complex systems that cannot be solved analytically. Grounded in probabilistic principles, Monte Carlo simulation finds applications across various fields, including mathematics, finance, physics, biology, and telecommunications. It was originally developed during World War II by John von Neumann and Stanislaw Ulam to enhance decision-making in uncertain environments [94]. The main steps of the Monte Carlo simulation method are as follows:

1 *Definition of the Problem:* Clearly outline the problem in terms of random variables.
2 *Quantification of Probabilistic Characteristics:* Determine the probabilistic characteristics of all random variables based on historical data and/or expert opinions.
3 *Random Generation of Variable Values:* Generate random values for each variable.
4 *Deterministic Evaluation:* Assess the problem using each set of values generated for the random variables.
5 *Extraction of Probabilistic Information:* Derive probabilistic insights from the generated values.
6 *Evaluation of Simulation Accuracy:* Assess the accuracy and efficiency of the simulation process.

Monte Carlo simulation necessitates a substantial number of random samples to ensure or enhance the accuracy of results. By generating multiple possible outcomes that encompass all potential values for each modeled variable, this method provides a deeper understanding of result stability and effectively accounts for uncertainty—an essential factor in multi-criteria decision-making. Utilizing non-deterministic approaches, Monte Carlo simulation offers approximate solutions to complex systems beyond the reach of theoretical mathematics through the experimentation of random numbers (Of & Dgcum, 1989). Wicaksono et al. (2020) introduced an innovative methodology that incorporates both probabilistic and epistemic uncertainties stemming from the inherent fuzziness in human judgments within multi-criteria decision analysis, termed the Norm-dist Monte-Carlo Fuzzy Analytic Hierarchy Process (NMCFAHP). Additionally, Díaz et al. [53] combined Monte Carlo simulation with AHP and FAHP to identify suitable sites for wind farms in Spain, utilizing key decision criteria alongside the subjective judgments of decision-makers. Dahri & Abida [43] integrated AHP with Monte Carlo simulation to assess flood susceptibility in the Gabès basin, located in southeastern Tunisia. The steps of the Monte Carlo AHP are illustrated in Step 4 of Fig. 4, and Table 8 presents the results of the MC-FAHP.

### 2.9 Overlaying raster layer

The overall suitability score is determined by calculating the land suitability index according to Equation (1), where *W*_*i*_ is the ith criterion weight and *P*_*i*_ is the criterion score of the ith factor [38].

(1) $$S=\left(\sum W_{i}P_{i}\right)$$

Final map is obtained from Equation (2) by multiplying the map from Equation (1) by the constraint layer using the Grass-GIS "raster calculator" tool.

(2) $$SuitableMap=\left(\sum W_{i}P_{i}\right)*Constraint$$

### 2.10 Solar hydrogen production potential

Photovoltaic (PV) solar panels generate the direct current (DC) required for water electrolysis. Their well-established and versatile characteristics make them an ideal choice for this application. In this study, a mono-crystalline photovoltaic panel with a maximum power output of 252.84 W and an efficiency of 17 % was utilized [17]. The annual energy conversion of the photovoltaic panel can be calculated using Equation (3) [38,95]:

(3) $$E_{PV}=G*A*SC*\eta_{PV}$$

Where E_PV_ is the energy produced by the photovoltaic panel (kWh/year), G represents the global horizontal irradiation (GHI) in kWh/m^2^/year, η_PV_ is the reference efficiency of the module (16 %), SC is the occupancy factor (70 %) and A is the total module area (m^2^). Power conditioning units in photovoltaic systems are employed to enhance electrical performance, modifying and regulating electricity production to achieve efficiencies of up to 97 %. However, typical efficiency rates reported in relevant literature hover around 85 %.

Several electrolysis technologies are available, with alkaline, proton exchange membrane (PEM), and solid oxide electrolysis cells (SOEC) being the most advanced and widely used. PEM electrolysis stands out as a leading method for producing clean, renewable hydrogen, supported by numerous studies [1,2,7,8,9,10,11,12,13,14,15,16,17,18,19,58,60,62,66]. This study selects the PEM electrolysis system due to its numerous advantages: high efficiency in converting electrical energy into hydrogen; durability and reliability for long-term use; compatibility with various renewable energy sources, including solar, wind, and hydro; and the ability to produce hydrogen at high pressure (1.2 bar), thereby reducing the need for gas compression and facilitating pipeline and storage integration. Hydrogen production is evaluated using specific assumptions, values, and equations. To produce 1 kg of hydrogen, 53 kWh of energy is required, assuming an energy efficiency of 75 % based on hydrogen's gross calorific value (GCV). The mass of hydrogen produced, denoted as M_H2_ (kg/km^2^/year), is calculated using Equation (4) [2,96]:

(4) $$\begin{array}{c} M_{H2}=\frac{ET*\eta_{ele}}{{HHV}_{H2}} \end{array}$$

Where MH₂ represents the annual hydrogen conversion (kg/year), E_PV_ is the conversion of solar electric source (kWh/year), η_ele_ is the efficiency of the electrolysis system (75 %) and HHV_H2_ is the higher calorific value of hydrogen (39.4 kWh/kg).

## 3 Results and discussion

### 3.1 Results of AHP, FAHP and MC-FAHP approaches

**Fig. 3:**
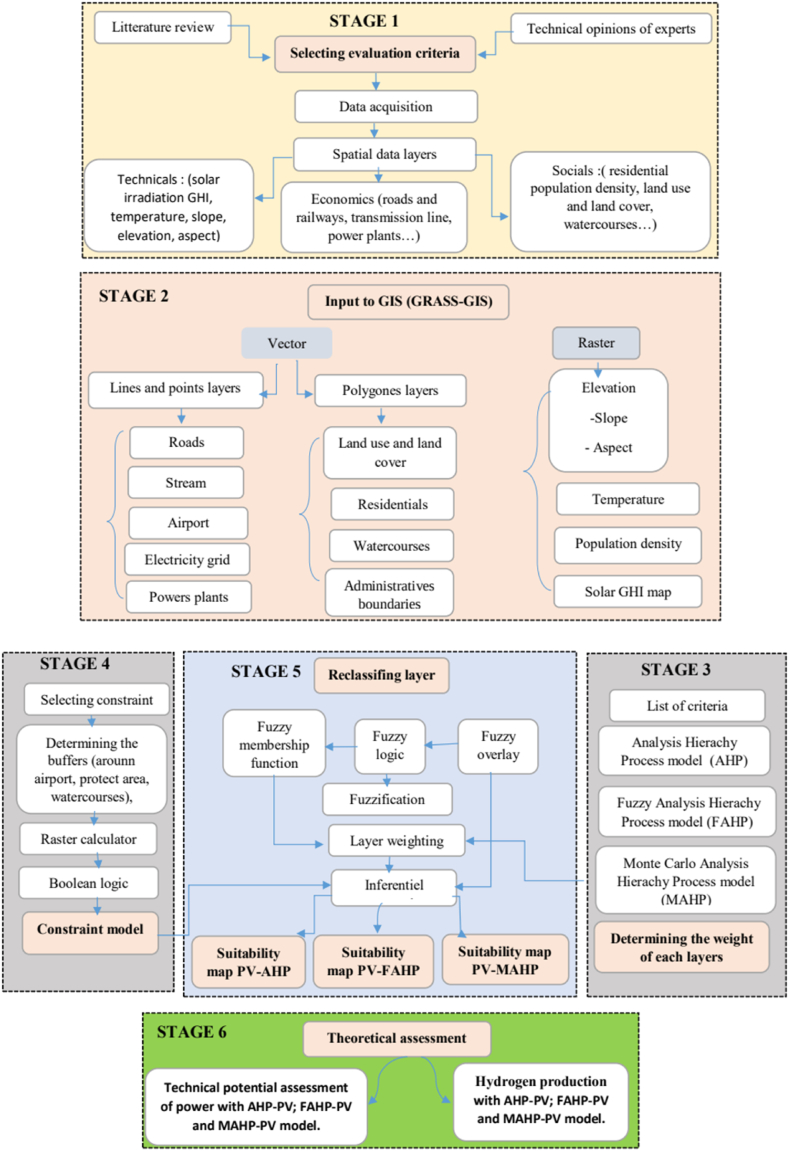
Methodological approach for Solar-Hydrogen conversion site selection.

Suitability maps for converting solar energy into hydrogen were developed using a combination of AHP, FAHP, and MC-FAHP methodologies to calculate criteria weights, integrated with GIS tools. These approaches provided a robust framework for site selection by evaluating technical, economic, and social criteria. Table 15 presents data from expert input, which was essential for constructing the comparison matrix, a key step in advancing this research. Fig. 8 displays the criteria weights derived from the AHP, FAHP, and MC-FAHP methods, while Fig. 3 outlines the overall methodological approach for identifying optimal Solar-Hydrogen conversion sites. Fig. 6 shows the resulting adequacy maps based on these methodologies. In addition, Table 2 lists the spatial and thematic layers utilized, and Table 3 outlines the specific criteria and constraints applied during the analysis. Table 9 summarizes the weights assigned to the technical, economic, and social criteria for solar PV-hydrogen farms across the AHP, FAHP, and MC-FAHP methods. The site evaluation was based on a rating scale from 0 to 4, where higher values indicated more favorable conditions for solar hydrogen production, and zero indicated unsuitable areas. The final suitability maps were classified into five categories: “0” (unsuitable), “1” (less suitable), “2” (suitable), “3” (highly suitable), and “4” (most suitable). This classification was performed by multiplying the values of each criterion by their corresponding weight and summing the results to produce the final map. A quantitative analysis of the suitability zones was carried out, with the results summarized in Table 12. This process involved converting the five suitability classes into polygonal layers for further assessment. The results indicate that approximately 42.40 % of the total area was classified as unsuitable for solar hydrogen production in all three methods (AHP, FAHP, and MC-FAHP). The "suitable" category covers around 15.26 %, 13.74 %, and 13.66 % of the total area for AHP, FAHP, and MC-FAHP, respectively. The "highly suitable" areas make up 38.83 %, 40.15 %, and 40.28 %, while the "most suitable" areas account for 3.50 %, 3.71 %, and 3.66 % across the three methods. Interestingly, no areas were classified as “less suitable” in any of the models. Fig. 9, Fig. 10, and Fig. 11 provide a visual comparison of the surface area, theoretical solar power potential, and potential hydrogen production across the suitability categories for the AHP, FAHP, and MC-FAHP methods. These figures highlight the consistency of the results between the three approaches, with slight variations in the distribution of “suitable” and “highly suitable” areas, reflecting the ability of FAHP and MC-FAHP to manage uncertainties and refine decision-making outcomes.

**Fig. 4:**
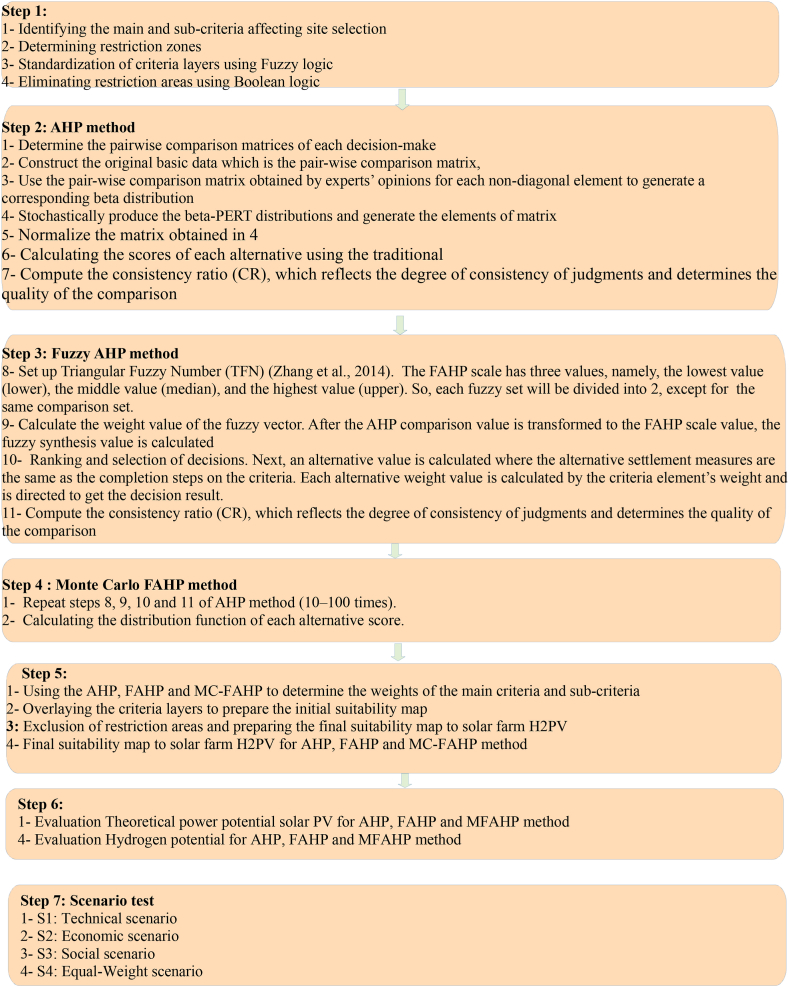
Methodology of study (the steps for selecting the suitable area of solar farm H2PV).

**Fig. 5:**
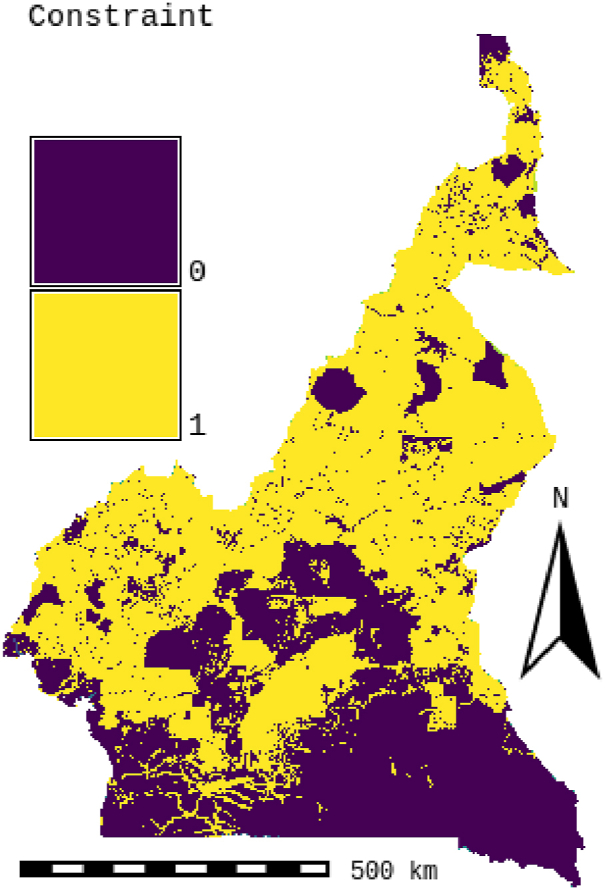
Constraint layer.

**Fig. 6:**
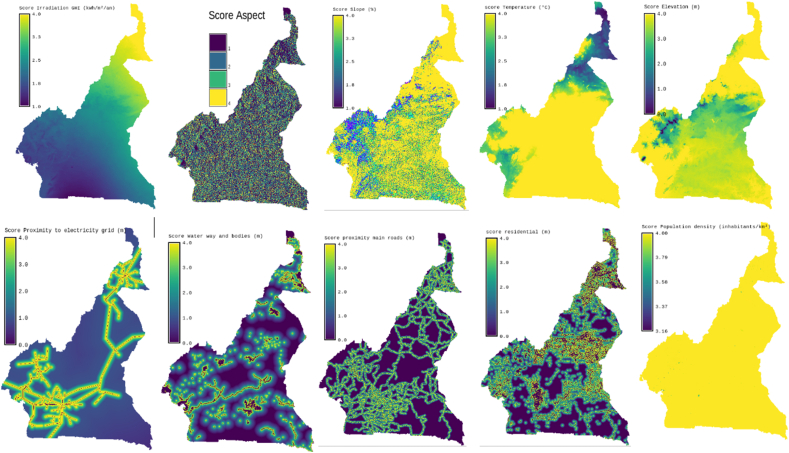
Score layers of location factors for solar H2PV farms.

**Fig. 7:**
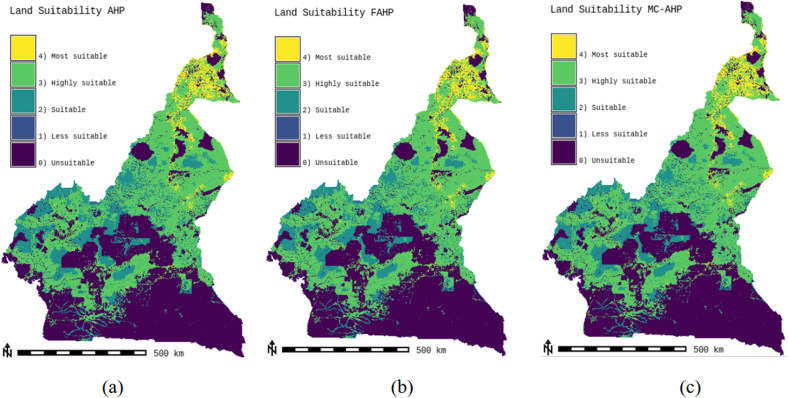
Land suitability of hybrid solar H2PV farms for AHP (a), FAHP (b) and MC-FAHP (c) methods.

**Fig. 8:**
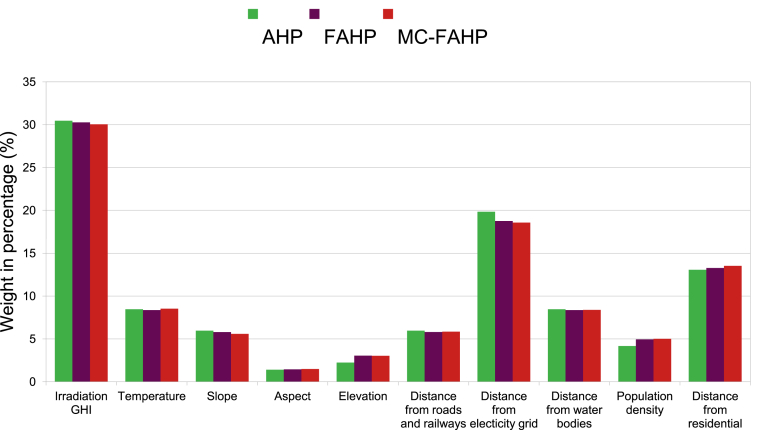
Weight of criteria in % for AHP, FAHP and MC-FAHP methods.

**Table 1:** Summary of criteria and siting methods considered in the studied references.

N°	Study	Case study region	Applied technique	Purpose/target	Siting criteria	Criteria
1	(Messaoudi et al., 2019) [9]	Algeria	GIS and AHP	GIS based multi-criteria decision making for solar hydrogen production sites selection in Algeria	(Hydrogen demand, Potential hydrogen production, DEM, Slope, P.T. roads, P.T. Railways, P.T. Power line)	07
2	(S. S. Hosseini Dehshiri et al.) [21]	Yazd province in Iran	GIS and fuzzy SWARA	A novel four-stage integrated GIS based fuzzy SWARA approach for solar site suitability with hydrogen storage system	P.T. the settlements, P.T. roads, P.T. transmission lines, Slope, Aspect, GHI, Temperature, Dust optical thickness, Dust deposition rate	09
3	(Taoufik & Fekri, 2023) [16]	Souss-Massa Region in Morocco	GIS, AHP and FAHP	A GIS-based multi-criteria decision-making approach for site suitability analysis of solar-powered hydrogen production in the Souss-Massa Region, Morocco	Hydrogen production potential, Hydrogen demand, Groundwater availability, Slope, Elevation; Roads, Industrial zones, Agriculture lands, The power grid.	09
4	(Amrani et al., 2024) [12]	Morocco	GIS and AHP	An AHP-GIS combination for site suitability analysis of hydrogen production units from CSP &PV solar power plants in Morocco	GHI, DNI, Slope, P.T. Cities, P.T. electricity Grid, P.T. the Ports, P.T. main roads, P.T. Gas pipelines, P.T. Phosphate industry, P.T. Railways, Population density, P.T. Dams, P.T. water ways, P.T. aquifers, P.T. Sea shores	14 (solar PV) 14 (solar (CSP)
5	(Ali et al., 2022) [2]	Southern Thailand	GIS and AHP	Suitable Site Selection for Solar‐Based Green Hydrogen in Southern Thailand Using GIS‐MCDM Approach	Hydrogen generation potential, P.T. major urban centers, Slope Elevation, P.T. road, P.T. transmission lines, P.T. waterbodies, P.T. protected areas, P.T. residential areas, Land type used	10
6	(Karipoğlu et al., 2022) [8]	Turkey	GIS	GIS-based optimal site selection for the solar-powered hydrogen fuel charge stations	GHI, Slope, Residential areas, Energy transmission lines, Main Roads, Temperature, Agricultural Areas, Designated Regions,	8
7	(Almasad et al., 2023) [39]	Saudi Arabia	GIS and FAHP, PROMETHEE II	Site suitability analysis for implementing solar PV power plants using GIS and fuzzy MCDM based approach	Global Horizontal irradiance, Average temperature, Precipitation, Air Pressure, Surface Albedo, Relative Humidity, Slope, Aspect, P.T. grids, P.T. power Lines, P.T. highways, P.T. major cities	12
8	(Y. Noorollahi et al., 2022) [38]	Khuzestan province	GIS, Fuzzy-Boolean logic and AHP	A framework for GIS-based site selection and technical potential evaluation of PV solar farm using Fuzzy-Boolean logic and AHP multi- criteria decision-making approach	GHI, Sunshine Duration, Temperature, Relative Humidity, Wind Speed, P.T. Power Line, P.T. Road, P.T. Substation, P.T. Urban Area, P.T. Rural Area, Slope, Aspect, Elevation, Land Use	14
9	(Saeidi et al., 2023) [30]	Tehran province, Iran	GIS, Fuzzy-Boolean logic and FUZZY-TOPSIS	FUZZY-TOPSIS method for defining optimal parameters and finding suitable sites for PV power plants	Forests-gardens-farms, Energy intake, Slope, Fault and mine, Small population center, Big population center, River, Lake, Power lines, freeway, Highway, Asphalt road, Railway, Military airport, Passenger airport, Dam, Industry	17
10	(Demir et al., 2023) [27]	˙Izmir, Türkiye	GIS, AHP and OBSG	A novel method for the site selection of large-scale PV farms by using AHP and GIS: A case study in ˙Izmir, Türkiye	Solar radiation rate, Land use, Slope	3
11	(Raza et al., 2023)[46]	Pakistan	GIS and AHP	Site suitability for solar and wind energy in developing countries using combination of GIS- AHP; a case study of Pakistan	GHI, Temperature, Slope, Land Aspects, Land Cover, P.T. Roads, Wind Speed, Wind Power Density, Elevation P.T. Cities, P.T. Cities, P.T. Power lines, P.T. Power lines	10 (solar) and 8 (wind)
12	(Hooshangi et al., 2023)[25]	Iran	GIS, TNF-TOPSIS and Fermatean Fuzzy TOPSIS	Evaluation of potential sites in Iran to localize solar farms using a GIS-based Fermatean Fuzzy TOPSIS	Land cost, Energy consumption, Land use, Land orientation, Aspect, Slope, F.T. active faults, F.T. dusty areas, Vegetation cover (NDVI), Temperature, Sunshine hours, Rainfall, Annual global solar irradiance, F.T. main road, F.T. transmission lines, F.T. rivers, F.T. synoptic stations or meteorological stations, Population, F.T. population centers	18
13	(Kocabaldır & Yücel, 2023) [28]	Turkey	GIS and AHP	GIS-based multicriteria decision analysis for spatial planning of solar photovoltaic power plants in Çanakkale province, Turkey	GHI, Duration of sunshine, Average annual temperature, Average annual relative humidity, Slope, Aspect, P.T. water resources, P.T. highways, P.T. transmission lines, P.T. substations, P.T. residential areas, P.T. fault lines, P.T. pit and quarry sites	13
14	(Mokarram et al., 2020) [47]	–	GIS, fuzzy-AHP and fuzzy-ANP	A novel optimal placing of solar farms utilizing multi-criteria decision-making (MCDA) and feature selection	GHI, Temperature, Slope, Elevation, Land use, P.T. Roads, P.T. residentials, P.T. Power lines, Cloudy days, Relative humidity, Dusty days	11
15	(Fotsing et al., 2020) [48]	Cameroon	GIS and Boolean method	GIS-based assessment of photovoltaic (PV) and concentrated solar power (CSP) generation potential in Cameroon using a Boolean method	GHI, Slope, Temperature, P.T. roads, P.T. the power grid, P.T. residential, P.T. streams, P.T. rivers, Elevation, Protect area, Population density, Plant required area	12
16	Colak, H et al., 2019) [49]	Malatya Province, Turkey	GIS and AHP	Optimal site selection for solar photovoltaic (PV) power plants using GIS and AHP: A case study of Malatya Province, Turkey	GHI, P.T. Fault lines, P.T. roads, P.T. the power grid, P.T. residential, P.T. Natural gas lines, P.T. Transformer centers, P.T. Lakes and dams, Aspect, P.T. Natural gas lines, Land cover	10
17	(Shahid Ali et al., 2018) [50]	Songkhla, Thailand	GIS and AHP	GIS based site suitability assessment for wind and solar farms in Songkhla, Thailand	GHI, Wind speed, Slope, Elevation, Land use, P.T. urban, P.T. Rural area, P.T. to Water Line, P.T. Forest, P.T. airport, P.T. Road, P.T. transmission line, Farm required area	12 (solar) 12(wind)
18	(Alisa Yushchenko et al., 2017) [51]	West Africa	GIS and AHP	GIS-based assessment of photovoltaic (PV) and concentrated solar power (CSP) generation potential in West Africa	GHI, DNI, Population density, P.T. settlements, P.T. roads, P.T. the power grid,	5 (solar PV) 5(solar CSP)
19	(Gacu et al., 2023) [52]	Sibuyan Island of Romblon, Philippines	GIS and AHP	Based Analytic Hierarchy Process (AHP) for Solar Power Exploration	Temperature, Solar Photovoltaic Power Output, DNI, GHI, DHI, P.T. Roads P.T. Transmission Lines, P.T. Coastal Areas, Elevation, Slope, Land Cover, Relative Humidity, Average Annual Cloud Cover, Flood Susceptibility, Landslide Susceptibility	15
20	(Díaz et al., 2022)[53]	Spain	GIS, Monte Carlo AHP AHP, and FAHP	Application of Monte Carlo and Fuzzy Analytic Hierarchy Processes for ranking floating wind farm locations	Wind velocity, Wind potential, Water depth, Wave conditions, Marine currents, Temperature, Technical feasibility, Sufficient study times, P.T. local electrical grid, P.T. coastal facilities, P.T. shore, P.T. residential areas, P.T. maritime routes, P.T. underwater lines, P.T. marine recreational activities, P.T. airport, P.T. protected areas, P.T. migratory birds' paths, P.T. migratory marine life paths, Area of the territory, P.T. the area of electricity demand, Population served, Multiple resources	23

P.T.: Proximity to.

GIS: Geographical Information Systems.

Fuzzy-ANP: Fuzzy Analytical Network Process.

Monte Carlo AHP: Monte Carlo Analytical Hierarchy Process.

TOPSIS: Technique for Order of Preference by Similarity to Ideal Solution.

SWARA: Step-wise Weight Assessment Ratio Analysis.

OBSG: Optimality-Based Site Growing.

PROMETHEE: Preference Ranking Organization METHod for Enrichment of Evaluations.

MCDM: Multi-Criteria Decision-Making.

Fuzzy-AHP: Fuzzy Analytical Hierarchy Process.

AHP: Analytical Hierarchy Process.

DHI: Diffuse Horizontal Irradiation.

GHI: Global Horizontal Irradiation.

DNI: Direct Normal Irradiation.

**Table 2:** Description of the spatial and thematic layers used in this study.

Spatial data layer and unit	Types (format)	Scale/resolution	Sources
Administrative boundaries of Cameroon (regions, departments, districts) Map	Vector (shapefile)	Polygons	Downloadable from: https://gadm.org [69]
Annual (GHI) in kWh/m^2^	Raster	(1∗1 km^2^)	Downloadable from: (https://solargis.com) [70]
Temperature (°C)	Raster	(1∗1 km^2^)	Downloadable from: (https://solargis.com) [70]
Map of population density in Cameroon	Raster	(1∗1ha)	**Downloadable from:**http://www.worldpop.org.uk/data [71]
Map of Cameroon Power lines	Vector (shapefile)	Lines	**Downloadable from:**http://databank.banquemondiale.org/data/reports.aspx?source=2&country=CMR [72]
Hydrological map of Cameroon (streams, navigable waters, rivers, rivers, wetlands, reservoirs …).	Vector (shapefile)	Polygons	Downloadable from: OSM,2023 https://www.openstreetmap.org/#map=6/7.402/12.343 [73]
Map of land use and land cover in Cameroon Map	Vector (shapefile)	Polygons	Downloadable from: OSM,2023 https://www.openstreetmap.org/#map=6/7.402/12.343 [73]
Map of the road network (inter_state, primary, secondary roads …) in Cameroon.	Vector (shapefile)	Lines	Downloadable from: OSM,2023 [90] https://www.openstreetmap.org/#map=6/7.402/12.343 [73]
Map of elevation in Cameroon	Raster	(1∗1 km^2^)	**Downloadable from:**https://globalwindatlas.info/ [67]
Map of Cameroon airport	Vector (text format csv) Geometry	Points	**Downloadable from:** ADC (Cameroon airport),2023 [68]

**Table 3:** Criteria and constraints.

Category	Description
**Constraint**	For the optimal identification of sites for solar power plant installation, it is crucial to consider multiple restrictive criteria that prioritize environmental protection, biodiversity conservation, and population safety. Areas deemed unsuitable due to technical, economic, or socio-environmental limitations will be excluded from the initial mapping using geographic information systems (GIS) [8,9,16,45,48,75]. Based on a detailed analysis of the study area's geography, a comprehensive literature review, and expert recommendations, specific exclusion criteria have been established, as outlined in Table 10. Zones impacted by infrastructures such as transmission lines, roads, cities, and their surrounding buffer areas will be excluded from consideration. Additionally, areas with steep slopes (> 20 %), altitudes below 50 m or above 2000 m, and locations more than 10 km from power lines or roads, or over 45 km from residential areas, will also be removed from the potential site selection. A 2,500-m buffer zone around airports is included to minimize the negative impacts of H2PV solar power plants on aviation, such as visual distractions for pilots, risks of accidents, and potential electromagnetic interference. Fig. 2-h illustrates the distribution of airports in Cameroon. It is essential to conduct a detailed assessment of available land when selecting sites for H2PV solar power plants. These plants should be situated outside of protected areas to minimize their environmental impact. Protected areas include parks, forests, and nature reserves, all of which are considered in this analysis. Table 10 provides a summary of the excluded zones, their buffer distances, and the maximum allowable distances from key infrastructure. The final constraint map is generated using a Boolean method through GIS, as shown in Fig. 5. In this map, areas deemed unsuitable are assigned a value of "0," while suitable areas are marked with a value of "1." This approach helps in visually distinguishing the potential sites for solar power plants, ensuring that only environmentally and technically feasible locations are considered for development.
**Technical**	**Solar Resource GHI (P1)**: Global Horizontal Irradiance (GHI) is a critical factor when selecting locations for solar photovoltaic (PV) installations intended for hydrogen production [16,39,52,76,77,78,79,80]. GHI represents the total solar energy received by a horizontal surface at ground level, which includes direct sunlight as well as diffuse solar radiation. The higher the GHI, the greater the potential for electricity generation, which can subsequently be used to produce hydrogen via water electrolysis. Therefore, GHI is a key consideration when determining the optimal location for solar PV plants dedicated to hydrogen production. The National Renewable Energy Laboratory (NREL) classifies solar irradiation into four categories: moderate (<4 kWh/m^2^/day), good (4–5 kWh/m^2^/day), very good (5–6 kWh/m^2^/day), and excellent (>6 kWh/m^2^/day). To evaluate GHI levels for site selection, an ascending linear fuzzy function was applied [24,76], with parameters outlined in Table 7 of the study. Solargis provides high-resolution raster data (1 km x 1 km) for GHI in Cameroon, with values ranging from 1,200 kWh/m^2^ to 2,193 kWh/m^2^ [70]. These values are categorized into five suitability classes using fuzzy logic: unsuitable (0), less suitable (1), suitable (2), very suitable (3), and highly suitable (4), as shown in Table 11. Fig. 2-a presents the distribution of GHI across Cameroon, illustrating areas with varying levels of solar potential.
	**Temperature (P8)**: Numerous studies emphasize the importance of temperature in selecting an H2PV solar power plant site due to its significant impact on efficiency [29,47,65,81,82,83]. Higher temperatures can reduce the efficiency of solar panels, which in turn affects hydrogen production. When panels overheat, they lose electrical output, diminishing overall plant performance. To account for this, a fuzzy declining linear function was applied to the temperature data, with its parameters detailed in Table 7. The temperature map, created using a 1 km x 1 km resolution Solargis raster [70], shows temperatures across Cameroon ranging from 7.8 °C to 28.6 °C (Fig. 2-b). The resulting fuzzy maps were classified according to the criteria in Table 11.
	**Slope (P7)**: Slope is another critical factor in the site selection process for solar PV plants dedicated to hydrogen production, as it directly impacts panel efficiency [2,8,9,15,16,48,75]. Steep slopes can cast shadows, obstruct sunlight, and hinder panel orientation and tilt, ideally due south at a 35° angle. Additionally, installation and maintenance costs increase in rugged or mountainous areas. Generally, acceptable slopes for solar installations range from 5 % to 20 %, depending on the reference [2,8,9,16,48,75], and this study sets the maximum threshold at 20 %. The slope map (Fig. 2-d) was created using World Wind Atlas data [67], with values between 0 % and 70 %. A fuzzy descending linear function was applied for slope classification, with parameters detailed in Table 7.
	**Aspect (P5)**: The orientation of photovoltaic panels plays a crucial role in optimizing hydrogen production and cost efficiency [24,26,76]. The aspect influences the amount of sunlight captured, directly affecting panel performance. For optimal exposure, the ideal orientation is due south with a 35° inclination. In this study, the aspect map was derived from the digital elevation model (DEM) and categorized into nine classes: flat (-1), north (0–22.5° and 337.5–360°), northeast (22.5–67.5°), east (67.5–112.5°), southeast (112.5–157.5°), south (157.5–202.5°), southwest (202.5–247.5°), west (247.5–292.5°), and northwest (292.5–337.5°) [24]. In Fig. 2-c, the aspect factor is reclassified as follows: flat areas are "unsuitable," while south-facing areas are considered "mostly suitable" [76], as detailed in Table 11.
	**Elevation (P6)**: Although elevation has a less direct effect compared to factors like solar irradiation and temperature, it still influences site selection for H2PV plants. Higher elevations often receive more solar radiation and experience lower temperatures, which can enhance panel efficiency [2,47,76,85,86]. However, altitudes below 50 m or above 2000 m were deemed "unsuitable" due to increased costs and logistical challenges [84]. The elevation map (Fig. 2-e), generated from Global Atlas Wind data [67], shows altitudes in Cameroon ranging from -26 m to 4024 m. A fuzzy descending linear function was used to process elevation data, with classification based on Table 11.
**Economic**	**Distance from Main Roads, Highways, and Railways (P10)**: Proximity to roads and railways is essential for the installation and maintenance of H2PV plants, as it facilitates transportation and access [1,2,8,9,13,16,48]. A distance of 500 m from major transport networks ensures safety, while a 10 km radius around roads is considered suitable for installations. Data on roads and railways, sourced from OpenStreetMap (OSM) [73], were used to create a proximity map (Fig. 2-f), processed with a fuzzy triangular function, and classified as per Table 11.
	**Distance from Electricity Grid (P2)**: Proximity to the electrical grid is a critical factor for integrating solar energy into the national grid, reducing connection costs, and ensuring stable power distribution [1,2,8,9,11,12,13,16,38]. In this study, a 10 km radius from the grid was considered ideal for H2PV plant installations, with a safety margin of 500 m to minimize risks. The electricity network data were sourced from the World Bank and validated by SONATREL (National Electricity Transport Company of Cameroon) [72]. Fig. 2-k shows the grid proximity, and the fuzzy map was classified as per Table 11.
	**Distance from Waterways and Water Bodies (P4)**: Water proximity is vital for H2PV solar plants, as water is needed for electrolysis and cooling [2,8,9,13,48,60,74]. A 45 km radius around water sources was considered optimal, though flood risk and other hazards necessitate a safety buffer of 500 m [8,9,16,45,75]. Data on waterways were sourced from OSM [73], with the distance map processed using a fuzzy linear function (Fig. 2-g), classified according to Table 11.
**Social**	**Population Density (P9)**: Population density is crucial for determining energy demand, infrastructure availability, and potential community impacts [3,76]. Regions with higher population densities are more likely to benefit from H2PV solar plants, although such installations could impact local communities through noise, visibility, and land use. Data from WorldPop [71] were analyzed using an increasing linear fuzzy function, with maps reclassified as per Table 11. Fig. 2-h shows population density in Cameroon, ranging from 0 to 1440 inhabitants per km^2^.
	**Distance from Residential Areas (P3)**: Proximity to residential areas can simplify energy distribution but may also result in community disruptions, such as noise or land use conflicts [2,8,9,13,15,16,48,60]. A 15 km radius around residential areas was deemed suitable, with a safety buffer of 1000 m [13]. Data from OSM [73] were used to create a distance map, processed with a trapezoidal fuzzy function. Fig. 2-i illustrates residential areas, with classification as per Table 11.
	**Land Requirements**: Grid-connected solar PV systems require a minimum of 0.4 km^2^ of land [77]. Areas smaller than 0.4 km^2^ were excluded from the final map of eligible sites.

**Table 4:** Fuzzy membership functions used in the study.

Function type	Mathematical equation	Fuzzy function figures
Ascending	$\begin{array}{c} Linear\left(a,b\right)=\left\{\begin{array}{c} 0ifx\leq a\\ \frac{x-a}{b-a}ifa<x<b\\ 1ifx\geq b \end{array}\right\} \end{array}$ $\begin{array}{c} Linear\left(a,b\right)=\max\left[\min\left(\frac{x-a}{b-a},1\right),0\right] \end{array}$	
Descending	$\begin{array}{c} Linear\left(a,b\right)=\left\{\begin{array}{c} 1ifx\leq a\\ \frac{x-b}{a-b}ifa<x<b\\ 0ifx\geq b \end{array}\right\} \end{array}$ $\begin{array}{c} Linear\left(a,b\right)=\max\left[\min\left(\frac{x-b}{a-b},1\right),0\right] \end{array}$	
Triangular	$\begin{array}{c} Triangular\left(a,b,c\right)=\left\{\begin{array}{c} 0ifx\leq a\\ \frac{x-a}{b-a}ifa<x<b\\ \frac{c-x}{c-b}ifb\leq x\leq c\\ 0ifx\geq c \end{array}\right\} \end{array}$ $\begin{array}{c} Triangular\left(a,b,c\right)=\max\left[\min\left(\frac{x-a}{b-a},\frac{c-x}{c-b}\right),0\right] \end{array}$	
Trapezoidal	$\begin{array}{c} Trapezoidal\left(a,b,c,d\right)=\left\{\begin{array}{c} 0ifx\leq a\\ \frac{x-a}{b-a}ifa<x<b\\ 1ifb\leq x\leq c\\ \frac{d-x}{d-c}ifc<x<d\\ 0ifx\geq d \end{array}\right\} \end{array}$ $\begin{array}{c} Trapezoidal\left(a,b,c,d\right)=\max\left[\min\left(\frac{x-a}{b-a},1,\frac{d-x}{d-c}\right),0\right] \end{array}$

**Table 5:** The fundamental scale of absolute numbers (Saaty's 9-Point Weighting Scale).

Intensity of Importance	Definition
1	Equally Important
3	weakly important
5	Fairly important
7	Strong important
9	Absolutely important
2, 4, 6, 8	Intermediate value between above scale values

**Table 6:** Saaty's Scale for Decision-Making using Fuzzy-AHP.

Linguistic Scales for Importance	Triangular Fuzzy Number (TFN)	Triangular Fuzzy Reciprocal Numbers
Equally Important (EI)	(1,1,1)	(1,1,1)
Intermediate 1 (IM1)	(1,2,3)	(1/3,1/2,1)
Moderately Important (MI)	(2,3,4)	(1/4,1/3,1/2)
Intermediate 2 (IM2)	(3,4,5)	(1/5, 1/4, 1/3)
Important (I)	(4,5,6)	(1/6,1/5,1/4)
Intermediate 3 (IM3)	(5,6,7)	(1/7,1/6/1/5)
Very Important (VI)	(6,7,8)	(1/8,1/7,1/6)
Intermediate 4 (IM4)	(7,8,9)	(1/9,1/8,1/7)
Absolutely Important (AI)	(9,9,9)	(1/9,1/9,1/9)

**Table 7:** Membership functions and fuzzification parameters of criteria layers of PV-hydrogen.

Main criteria	Criteria	Fuzzy membership function type	Unit	a	b	c	d	
Technical	Solar ressource GHI (P1)	Linear Ascending	kWh/m^2^ year	1261	2202			[38] [47]
	Temperature (P8)	Linear Descending (2)	°C	7	28.6			[38]
	Slope (P7)	Linear Descending	%	2	6			[38] [13]
	Aspect (P5)	Five uniform grade						[84]
	Elevation (P6)	Linear Descending (1)	m	500	2000			[16]
Economic	Distance from roads and highways and railways (P10)	Triangular	m	500	2000	10000		[38] [13]
	Distance from electricity grid (P2)	Linear Descending (2)	m	500	30000			[38] [13]
	Distance from water way and bodies (P4)	Linear Descending	m	1000	45000			[16]
Social	Population density P9)	Linear Ascending	inhabitants/km^2^	0	1424			[84]
	Distance from residential (P3)	Trapezoidal	m	1000	2500	6500	15000	[38] [13] [38]

**Table 8:** Criteria and sub-criteria weights and standard deviation of PV-hydrogen site.

Criteria	Sub-criteria	Code	rank AHP	rank FAHP	rank MC-FAHP	Weight AHP	Weight FAHP	Weight MC-FAHP	Std AHP and FAHP	Std FAHP and MC-FAHP	std AHP, FAHP and MC-FAHP
Technical	Irradiation GHI	P1	1	1	1	30,46	30,27	30,04	0,13	0,16	0,2103
	Temperature	P8	4	4	4	8,45	8,36	8,53	0,06	0,12	0,085
	Slope	P7	6	6	6	5,96	5,79	5,58	0,12	0,15	0,1904
	Aspect	P6	10	10	10	1,41	1,44	1,48	0,02	0,03	0,0351
	Elevation	P5	9	9	9	2,24	3,04	3,03	0,57	0,01	0,4590
Economic	Distance from roads and railways	P10	6	7	7	5,96	5,8	5,85	0,11	0,04	0,0819
	Distance from electricity grid	P2	2	2	2	19,84	18,76	18,58	0,76	0,13	0,6815
	Distance from water bodies	P9	4	4	5	8,45	8,36	8,38	0,06	0,01	0,04726
Social	Population density	P4	8	8	8	4,16	4,94	5,01	0,55	0,05	0,4718
	Distance from residential	P3	3	3	3	13,07	13,27	13,52	0,14	0,18	0,2255

**Table 9:** AHP, FAHP and MC-AHP weight and CR of PV-hydrogen for technical, economic and social group.

Weight	Model	Technical	Economic	Social
	AHP	48,52 %	32,95 %	18,52 %
	FAHP	48,9 %	32,89 %	18,22 %
	MC-FAHP	48,67 %	32,6 %	18,5 %

**Table 10:** Restriction criteria from chosen solar PV-hydrogen.

Category	Boolean algebra restriction	Reference
Slope	x>20 %	[13]
Elevation	X<50m and x > 2000m	[16] [2]
Aspect	Flat	[84]
Buffer distance/distance to power grid	X < 500 m	[9] [2]
Buffer distance/distance to mains roads & highways and railways	x < 500 m and x > 10 km	[9] [2]
Buffer distance/distance to residential	X < 1 km and x> = 15 km	[13]
Land cover and land use	Not within land use	[9]
Buffer distance from airports	x < 2500 m	[38]
Buffer distance from water ways and bodies	x<1 km and >45 km	[9] [2]
Plant required area	x < 0,4 km^2^	[77]

**Table 11:** Suitability classification and weighting of the different scenarios of PV-hydrogen.

Score	Criteria	Technical					Economic			Social	
	Sub-criteria	Annual global horizontal irradiation (GHI)	Temperature [46]	Slope	Elevation [16] [2]	Aspect [84]	Proximity to roads, highways and railways	proximity from electricity grid [38]	proximity from residential [84]	Population density, Minimize density [84]	Proximity to Water ways and water bodies [2]
	Unit	kWh/m^2^ year	°C	%	m	–	m	m	m	inhabitants/km^2^	m
0	Unsuitable	<1300	–	>20	<50 and >2000	Flat	<500 and >10000	<500	<1000 and >15000	–	<1000 and >45000
1	Less suitable	1500–1300	27,5–28.6	10–20	1500–2000	N, NE, NW	8000–1000	>30000	10000–15000	1200–1440	20000–45000
2	Suitable	1750–1500	26,5–27,5	5–10	1000–1500	E, W	5000–8000	10000–30000	6500–10000	1000–1200	10000–20000
3	Highly suitable	2000–1750	25-26,5	2–5	500–1000	SE, SW	2000–5000	2500–10000	2500–6500	890–1000	5000–10000
4	Most suitable	>2000	<25	<2	<500	S	500–2000	500–2500	1000–2500	0–890	1000–5000
S0	Weighing sub-criteria	30,4	8,50	5,50	1,47	3,03	5,80	18,50	8,30	5,00	13,50
	Weighing criteria	48,9					32,6			18,50	
S1	Weighing sub-criteria	37,93	10,6	6,86	1,83	3,78	4,63	14,75	6,62	3,51	9,49
CR = 4.5 %	Weighing criteria	61					26			13	
S2	Weighing sub-criteria	16,17	4,52	2,92	0,78	1,61	10,88	34,54	15,58	3,51	9,49
CR = 4.5 %	Weighing criteria	26					61			13	
S3	Weighing sub-criteria	16,17	4,52	2,92	0,78	1,61	2,32	7,36	3,32	16,49	44,51
CR = 4.5 %	Weighing criteria	26					13			61	
S4	Weighing sub-criteria	10	10	10	10	10	10	10	10	10	10
–	Weighing criteria	50					30			20

**Table 12:** Statistical information of land suitability areas of solar farm H2PV.

Plage	Categories	Statistical	AHP-H2PV	FAHP-H2PV	MC-FAHP-H2PV	S1-H2PV	S2-H2PV	S3-H2PV	S4-H2PV
0–0	0 « unsuitable »	Areas in m^2^	242469932287	242469932287	242469932287	242469932287	242469932287	242469932287	242469932287
		pixels number	24264253	24264253	24264253	24264253	24264253	24264253	24264253
		%	42.40 %	42.40 %	42.40 %	42.40 %	42.40 %	42.40 %	42.40 %
0.0001–1	1 « less suitable »	Areas in m^2^	89935	–	–	0	698043111	0	0
		pixels number	9	–	–	0	69854	0	0
		%	0.00 %	–	–	0 %	0.12 %	0 %	0 %
1.001–2	2 « suitable »	Areas in m^2^	87275024898	78545788643	78104023105	56701378180	167748381435	132945345329	37298959432
		pixels number	8733715	7860170	7815962	5674174	16786779	13303998	3732551
		%	15.26 %	13.74 %	13.66 %	9.92 %	29.34 %	23.25 %	6.52 %
2.001–3	3 « highly suitable »	Areas in m^2^	222048408969	229573122642	230298995942	248938167994	141699543816	155641959166	275283284713
		Pixels number	22220647	22973654	23046293	24911537	14180041	15575275	27547924
		%	38.83 %	40.15 %	40.28 %	43.53 %	24.78 %	27.22 %	48.14 %
3.001–4	4 « most suitable »	Areas in m^2^	20020598712	21225830790	20941812962	23706425025	19204489644	40756588183	16765525775
		Pixels number	2003485	2124094	2095672	2372330	1921816	4078560	1677746
		%	3.50 %	3.71 %	3.66 %	4.15 %	3.36 %	7.13 %	2.93 %

**Table 13:** Theoretical power potential and hydrogen potential for the utility scale solar H2PV farms.

Methods	Categories /Parameters	Not Suitable (km^2^)	Less Suitable (km^2^)	Suitable (km^2^)	Highly Suitable (km^2^)	Most suitable (km^2^)	Total
	Mean GHI irradiation (kWh/m^2^/year)	–	1400	1750	2100	2200	
**AHP-H2PV**	Area in km^2^	242469,932	–	87275,0249	222048,409	20020,5987	571813,96
	Potential (TWh/year)	–	–	18175,024	55489,897	5241,393	78906,31
	Potential hydrogen (tonnes/year)	–	–	345971268	1056279773	99772704	1502023745
**FAHP-H2PV**	Area (km^2^)	242469,932	–	78545,7886	229573,123	21225,8308	571814,67
	Potential (TWh/year)	–	–	16357,160	57370,323	5556,923	79284,41
	Potential hydrogen (tonnes/year)	–	–	311367268	1092074685	105778982	1509220935
**MC-FAHP-H2PV**	Area (km^2^)	242469,932	–	78104,0231	230298,996	20941,813	571814,76
	Potential (TWh/year)	–	–	16265,163	57551,719	5482,567	79299,45
	Potential hydrogen (tonnes/year)	–	–	309616043	1095527648	104363578	1509507269

**Table 14:** Standard deviation of the theoretical electric and hydrogen potential for the AHP, FAHP and MC-FAHP.

	AHP	FAHP	MC-FAHP	St AHP and FAHP	St AHP, FAHP and MC-FAHP
Potential electricity in TWh/year	78906,31	79284,41	79299,45	267,357	222,765
Potential hydrogen (tonnes/year)	1502023745	1509220935	1509507269	5089181,854	4240374,64

**Table 15:** List of experts.

N°	Designation	Qualification	Age	Work experience	Department/Company
1	Professor	PhD	36	12	University of Dschang, Cameroon
2	Professor	PhD	50	25	University of Dschang, Cameroon
3	Assistant-Manager	PhD	43	20	Ministry of energy, Cameroon
4	Professor	PhD	58	35	ULB, Belguim
5	Energy expert	Graduate	42	18	Ministry of energy, Cameroon
6	Deputy-Manager	PhD	38	8	Solar Energy Technology, Cameroon
7	Professor	PhD	64	40	ULB, Belguim
8	Deputy-Director	PhD	52	25	ENEO, Cameroon
9	Engineer	Graduate	58	33	ULB, Belguim
10	Assistant-Manager	Graduate	35	11	SONATREL, Cameroon

Names of the experts not disclosed at the request of them.

**Fig. 9:**
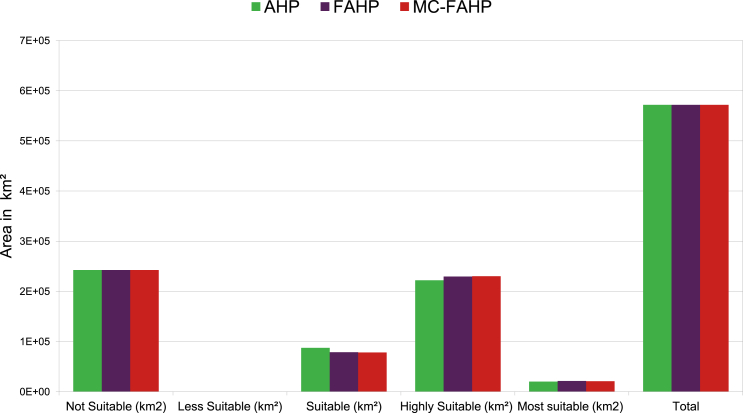
Area in km^2^ for AHP, FAHP and MC-FAHP methods.

**Fig. 10:**
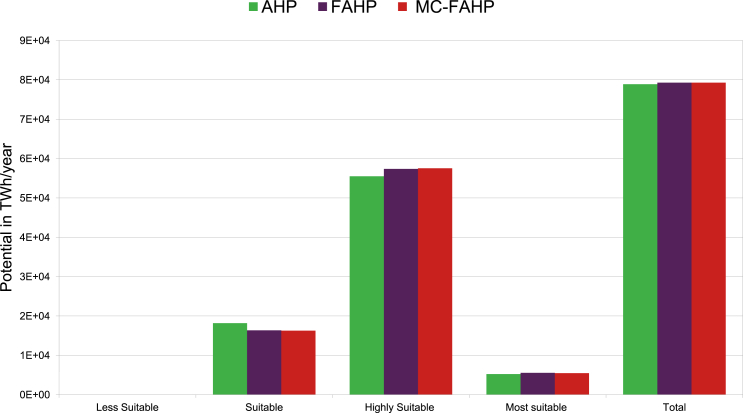
Theoretical power potential solar H2PV in TWh/year for AHP, FAHP and MC-FAHP methods.

**Fig. 11:**
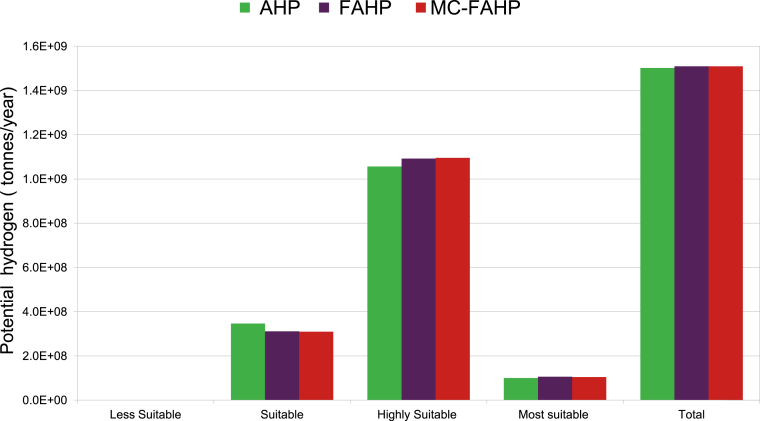
Potential hydrogen for AHP, FAHP and MC-FAHP methods.

### 3.2 Site suitability analysis using the AHP method

AHP method was applied to compare the suitability of different areas for solar-hydrogen conversion systems, using GIS tools to evaluate key criteria. The standardization of parametric datasets was crucial for ensuring uniformity in the assessment, allowing for the assignment of appropriate values to each criterion's class limits. Ten criteria were selected, grouped into three main categories: technical, economic, and social. Exclusion criteria were primarily based on environmental considerations, as detailed in Table 10.

AHP method was then used to assign weights to the factors, reflecting variations in their relative importance. The pairwise comparison results are presented in Table 8, and the validation of the eigenvectors was confirmed by calculating the Consistency Ratio (CR), which was less than 10 %, with a value of 9.72 %. Although discrepancies may arise from inconsistent comparisons between factors, the values for the Consistency Index (CI = 0.145) and CR suggest that the assigned weights were consistent and reliable. A comparative analysis of the criteria revealed that Global Horizontal Irradiation (GHI) is the most influential factor, with a weight of 30.46 %. Since hydrogen production via photovoltaic (H2PV) systems is directly proportional to the amount of solar irradiation, areas with higher GHI are particularly advantageous. The second most important criterion is the proximity to power lines, with a weight of 19.84 %. Maintaining a safety distance of more than 500 m from power lines is essential to mitigate risks, while close proximity ensures the efficient transport of electricity, contributing to plant stability.

Proximity to residential areas ranked third, with a weight of 13.07 %. Areas at least 15 km from residential zones, with a 1 km safety buffer, are ideal to optimize hydrogen distribution while ensuring public safety. The proximity to watercourses is the fourth key factor, weighing 8.45 %. Hydrogen production through electrolysis requires significant water resources, so locating H2PV plants within 45 km of rivers, while maintaining a 1 km flood safety buffer, is advantageous. Additionally, proximity to water simplifies cleaning solar panels, enhancing efficiency. Table 8 presents the weights of the remaining criteria. To visualize the results and identify optimal sites for H2PV units, these weights were incorporated into GIS software, enabling the creation of a suitability atlas. By assigning specific weights to each pixel in the sub-criteria maps, the GIS analysis generated a final suitability map that illustrates the overall potential of the land for solar hydrogen production facilities. The AHP-based suitability map is shown in Fig. 7-a. The results from these suitability maps suggest that Cameroon is highly favorable for establishing solar hydrogen production units using PV technology. Approximately 42.40 % of the country is deemed unsuitable for H2PV construction, while 57.6 % is suitable. The theoretical potential for electricity production is estimated at 78,906.31 TWh/year, and the hydrogen production capacity is projected to be around 1,502,023,745 tonnes/year. Further details on the distribution of suitable areas for H2PV plants are summarized in Table 12, and Table 13 provides calculations for the theoretical energy and hydrogen production potential for each assessment method.

### 3.3 Site suitability analysis for FAHP method

In the fuzzy approach, criteria weighting is conducted using a triangular evaluation method that utilizes triangular fuzzy numbers, as outlined in Table 6. The Fuzzy-AHP (FAHP) pairwise comparison matrix is derived from expert rankings, and from this matrix, the fuzzy geometric means for all parameters are calculated. These fuzzy geometric means are then used to assess the fuzzy criteria weights, which are subsequently defuzzified into corresponding non-fuzzy weights. The calculated weights for each criterion using the FAHP method are presented in Table 8. To validate the consistency of the results, the Consistency Ratio (CR) was computed, which, as in AHP, must remain below 10 %. The values of CI (Consistency Index = 0.099) and CR (6.67 %) confirm the consistency and reliability of the weightings derived from the FAHP method. While the priority order of the criteria remains the same as in AHP, the values differ slightly between the two methods. The weight assigned to solar irradiation, the most influential factor, is 30.27 % in FAHP compared to 30.46 % in AHP, resulting in a standard deviation of approximately 0.134. The second most influential factor, proximity to the electricity grid, has a weight of 18.76 % in FAHP and 19.84 % in AHP, with a standard deviation of about 0.764. For the third most important criterion, proximity to residential areas, the weight is 13.27 % in FAHP compared to 13.07 % in AHP, with a standard deviation of 0.141. The comparative standard deviations between FAHP and AHP are summarized in Table 8. To better visualize these results and pinpoint the optimal sites for H2PV hydrogen production units, the weights obtained via the FAHP method were integrated into GIS software, leading to the creation of a suitability atlas. In the GIS platform, based on the data in Table 11, a specific weight was assigned to each pixel of the sub-criteria maps. This process resulted in a final analysis map showing the total land capacity to accommodate solar hydrogen production facilities. The suitability map generated by the FAHP method is displayed in Fig. 7-b. The suitability maps confirm that Cameroon is a strategic location for establishing solar hydrogen production units utilizing photovoltaic technology. According to the FAHP method, approximately 42.40 % of the country's land is unsuitable for the installation of H2PV units, while 57.6 % of the land is deemed suitable. The theoretical electricity production potential is estimated at 79,284.41 TWh/year using the FAHP method, compared to 78,906.31 TWh/year with AHP, resulting in a standard deviation of 267.357. The hydrogen production capacity is projected to be approximately 1,509,220,935 tonnes/year with FAHP, compared to 1,502,023,745 tonnes/year with AHP, yielding a standard deviation of around 5,089,181.85 tonnes/year. Table 12, Table 13, and Table 14 summarize the results, including the surface distribution for each suitability category, the theoretical potential for electricity and hydrogen production, and the standard deviation of these potentials across the three methods (AHP, FAHP, and MC-FAHP). The results indicate that the FAHP method offers a slightly more optimistic estimation for energy and hydrogen production potential compared to AHP, reflecting its ability to better handle uncertainties in decision-making processes.

### 3.4 Site suitability analysis for MC-FAHP method

As FAHP is unable to manage an unbalanced weight scale, mode values are selected to replace mean values in the initial pairwise comparison matrices. The low, medium, and high fuzzy values are developed based on triangular fuzzy sets. In the final evaluation, FAHP utilizes a triangular distribution to generate 100 random numbers, contrasting with the normal distribution applied in the proposed MC-FAHP method. In both MC-FAHP, AHP, and FAHP, while the priority order of the criteria remains unchanged, their respective values vary. For example, the weight assigned to solar irradiation, the most influential factor, is 30.27 % for FAHP, 30.46 % for AHP, and 30.04 % for MC-FAHP, with a standard deviation of approximately 0.2103. Similarly, for proximity to the electricity grid, the second most influential criterion, the weight is 18.76 % for FAHP, 19.84 % for AHP, and 18.58 % for MC-FAHP, with a standard deviation of about 0.6815. For the proximity to residential areas, the third most important criterion, the weight is 13.27 % for FAHP, 13.07 % for AHP, and 13.52 % for MC-FAHP, with a standard deviation of approximately 0.2255. Detailed data on standard deviations between the three methods are presented in Table 8. To fully visualize these results and determine the optimal locations for H2PV hydrogen production units, the weights derived from FAHP were integrated into GIS software to create a suitability atlas. Using the data from Table 11, each pixel of the sub-criteria maps was assigned a specific weight. This procedure resulted in a final analysis map showing the overall capacity of the land to support solar hydrogen production facilities. The suitability map produced with the MC-FAHP method is displayed in Fig. 7-c. The suitability maps reveal that Cameroon represents a prime location for establishing solar hydrogen production units using photovoltaic (PV) technology. Overall, approximately 42.40 % of Cameroonian territory is considered unsuitable for H2PV installations, while about 57.6 % of the land is deemed suitable. The theoretical electricity production potential is estimated at 79,284.41 TWh/year using the FAHP method, compared to 78,906.31 TWh/year with AHP and 79,299.45 TWh/year with MC-FAHP, yielding a standard deviation of 222.765. Similarly, the hydrogen production capacity is estimated at 1,509,220,935 tonnes/year with FAHP, 1,502,023,745 tonnes/year with AHP, and 1,509,507,269 tonnes/year with MC-FAHP, with a standard deviation of approximately 4,240,374.64 tonnes/year.

The results for the distribution of surface areas across various suitability categories, as well as the theoretical potential for electricity and hydrogen production, are summarized in Table 12 and 13. Table 14 presents the standard deviation of the theoretical electricity and hydrogen potential across the AHP, FAHP, and MC-FAHP methods. These comparisons demonstrate that MC-FAHP tends to provide slightly more optimistic estimates of both electricity and hydrogen production capacity, highlighting its robustness in managing uncertainty in decision-making.

### 3.5 Comparative study between AHP, FAHP and MC-FAHP method

To conduct a comparative statistical analysis, we will scrutinize and contrast the outcomes of three methodologies utilized for evaluating areas apt for solar hydrogen production via photovoltaic panels (AHP-H2PV, FAHP-H2PV, and MC-FAHP-H2PV). This comparison will focus on three principal parameters displayed in the table: area in km^2^, energy potential (in TWh/year), and hydrogen production potential (in tons/year). For the "Not Suitable" category, all three methods concur on the same area (242,469.932 km^2^). In contrast, for the "Suitable," "Highly Suitable," and "Most Suitable" categories, significant discrepancies are observed among the methods. FAHP-H2PV allocates a more extensive area to the "Highly Suitable" category (229.573 km^2^) compared to AHP-H2PV (222.048 km^2^), while MC-FAHP-H2PV designates a considerable portion to the "Suitable" category, amounting to 230.299 km^2^. The aggregate energy potential shows minimal variation across the methods: AHP-H2PV (78.906 TWh/year), FAHP-H2PV (79.284 TWh/year), and MC-FAHP-H2PV (79.299 TWh/year). Nonetheless, the allocation of this energy potential differs notably. AHP-H2PV indicates a marginally greater potential in the "Suitable" category relative to the others, whereas FAHP-H2PV and MC-FAHP-H2PV assign a more substantial potential to the "Highly Suitable" category (57,370 TWh and 57,551 TWh, respectively). The overall hydrogen production potential is comparable across the methods, with MC-FAHP-H2PV holding a slight edge (1,509,507,269 tons/year), succeeded by FAHP-H2PV (1,509,220,935 tons/year) and AHP-H2PV (1,502,023,745 tons/year). Distribution-wise, FAHP-H2PV and MC-FAHP-H2PV exhibit a greater hydrogen potential in the "Highly Suitable" category compared to AHP-H2PV, which presents a marginally higher potential in the "Suitable" category.

In summary, the three methodologies reveal variations in the distribution of regions apt for solar and hydrogen production, primarily in the "Suitable" and "Highly Suitable" categories. Each method yields similar total energy potential estimates (approximately 79,000 TWh/year), yet they differ significantly in the potential distribution across categories. The total hydrogen production potential estimates are comparable, but their distribution by category differs among the methods. FAHP-H2PV and MC-FAHP-H2PV generally predict a greater potential in "Highly Suitable" areas.

FAHP-H2PV and MC-FAHP-H2PV seem more optimistic than AHP-H2PV about the contribution from "Highly Suitable" areas to solar and hydrogen production, though the overall outcomes of the three methods are similar. This indicates the proficiency of fuzzy approaches (FAHP and MC-FAHP) in managing uncertainties and variations when assessing criteria, as opposed to the traditional AHP method.

This article presents a comparative study of three decision-making methods: AHP (Analytic Hierarchy Process), FAHP (Fuzzy Analytic Hierarchy Process), and MC-FAHP (Monte Carlo FAHP). The objective is to evaluate the performance and precision of the MC-FAHP method in the decision-making process. The results show that all three methods generate a similar ranking of criteria, except in cases where the criteria have identical weights. In AHP and FAHP, criteria with the same weight are assigned the same rank, whereas MC-FAHP distinguishes between them. For example, the criteria "temperature" and "distance from waterways" both have a weight of 8.35 % and rank 4 in AHP and FAHP, but in MC-FAHP, they are ranked 4th and 5th, respectively. Additionally, criteria such as "slope" and "distance from main roads and railways," which share the same weight and rank (6th) in AHP, receive different weights in FAHP and MC-FAHP, though their ranks remain consistent at 6th and 7th, respectively. Analysis of Table 8, comparing the standard deviation between the FAHP and MC-FAHP methods, reveals that the use of a normal distribution in Monte Carlo simulations reduces the standard error in the final evaluation of weight means. This suggests that MC-FAHP yields fewer errors when addressing uncertainty and stochastic variability. The normal distribution used in MC-FAHP concentrates values around the mean, producing results close to the defuzzified mode and reducing the standard deviation error. In contrast, the triangular distribution in FAHP does not accurately reflect realistic standard deviation values, as it relies on a triangular fuzzy number instead of an initial standard deviation based on expert judgment. Furthermore, the Monte Carlo normal distribution in MC-FAHP generates a narrower confidence interval than the traditional FAHP method, enhancing the reliability of its results. Overall, the analysis concludes that the MC-FAHP method is more effective in managing uncertainty and producing realistic evaluation scores, making it a reliable decision support tool. Its ability to handle stochastic hazards with greater accuracy makes MC-FAHP a valuable advancement over traditional methods like AHP and FAHP.

### 3.6 Validation of sites planned for solar H2-PV

The solar power plants in Guider and Maroua serve as key validation points for the methodology presented in this study. As the only two large-scale solar installations currently connected to Cameroon's national electricity grid, they provide critical real-world data to assess the suitability of sites for solar energy development in the country. The precise geographical coordinates of these plants, located at 10.5956° N, 14.3247° E for Maroua (Extreme North) and 9.9333° N, 13.9478° E for Guider (North), are used for comparison in this research.

Upon plotting these coordinates on the final solar PV park suitability map, both plants are situated in areas classified as "highly suitable." This classification underscores the robustness of the proposed model in identifying prime locations for solar power generation. The "highly suitable" designation is a result of several favorable factors, most notably the region's high solar irradiance levels, which are essential for optimizing energy output. Meteorological data for these locations indicate significant annual solar irradiation, confirming that both Guider and Maroua have the potential to efficiently harness solar energy for large-scale electricity production. The operational success of these solar plants is further enhanced by the surrounding infrastructure. Proximity to roads and other transport networks facilitates the smooth delivery of equipment and materials, while their integration into the electricity grid ensures efficient power transmission. This infrastructure not only reduces logistics and transportation costs but also improves overall operational efficiency, contributing to the long-term viability of the installations. From an environmental perspective, preliminary assessments indicate minimal disruption to local ecosystems resulting from the construction and operation of these solar power plants. In fact, the plants are contributing to the country's environmental sustainability goals by significantly reducing greenhouse gas emissions. This reinforces the environmental benefits of investing in solar energy as a clean and renewable energy source in Cameroon. Fig. 13 illustrates the overlay of the Maroua and Guider power plants on the final solar PV suitability map, visually confirming the alignment between actual installations and model predictions. This overlay highlights the reliability of the site selection criteria used in this study, further validating the proposed approach for optimizing solar energy projects in Cameroon.

This research highlights the crucial role solar energy can play in Cameroon's energy transition, demonstrating the viability and potential of integrating solar power plants into the national grid. The use of Multi-Criteria Decision-Making (MCDM) techniques in conjunction with Geographic Information Systems (GIS) and Monte Carlo method enabled the identification of optimal locations for solar plant installations. Factors such as solar irradiance, topography, proximity to infrastructure, and environmental considerations were meticulously assessed. In addition to solar energy, the potential for generating green hydrogen using solar power was examined. As a clean energy carrier, hydrogen presents a promising solution for energy storage and reducing greenhouse gas emissions. Developing solar power plants and hydrogen production facilities could yield substantial economic benefits, including job creation, reduced dependence on imported fossil fuels, and improved access to reliable energy for local communities. However, the project also identified several challenges, including the need for accurate and up-to-date data, high initial investment costs, and existing regulatory barriers. Addressing these issues requires developing local expertise in energy project management and fostering policies that support renewable energy development. Moving forward, continued investment in research and development (R&D) in solar and hydrogen technologies is essential. Collaboration between the public and private sectors, with support from international organizations, will be critical to fully unlocking Cameroon's solar energy potential. This research lays a strong foundation for a sustainable energy transition in Cameroon, presenting both the opportunities and challenges associated with solar power plant adoption and green hydrogen production. It sets the stage for the expansion of renewable energy infrastructure, contributing to a cleaner, more resilient energy future.

The study provides key implications for Cameroon's energy policies:

1 **Optimized Site Selection**: Identifying optimal solar hydrogen production locations allows policymakers to prioritize high-potential areas, boosting renewable energy investments.
2 **Advanced Decision-Making**: The use of AHP, FAHP, and MC-FAHP highlights the value of incorporating advanced decision tools into energy planning, enhancing adaptability and resilience.
3 **Socio-Economic Considerations**: Policies should account for economic and social factors, such as job creation and regional equity, to maximize benefits from renewable energy projects.
4 **Sustainability**: The findings support long-term energy strategies focused on renewables, enhancing energy security and contributing to global climate goals.

### 3.7 Statistical analysis of sensitivity results

A sensitivity analysis was conducted to evaluate the robustness of the proposed model by altering the weights of input criteria and observing their impact on the final suitability map. The sensitivity test was structured into four scenarios: S1 (Technical criteria-focused); S2 (Economic criteria-focused); S3 (Social criteria-focused); S4 (Equally weighted). The base scenario (S0), derived from the MC-FAHP method, serves as the reference for comparison.

**Fig. 12:**
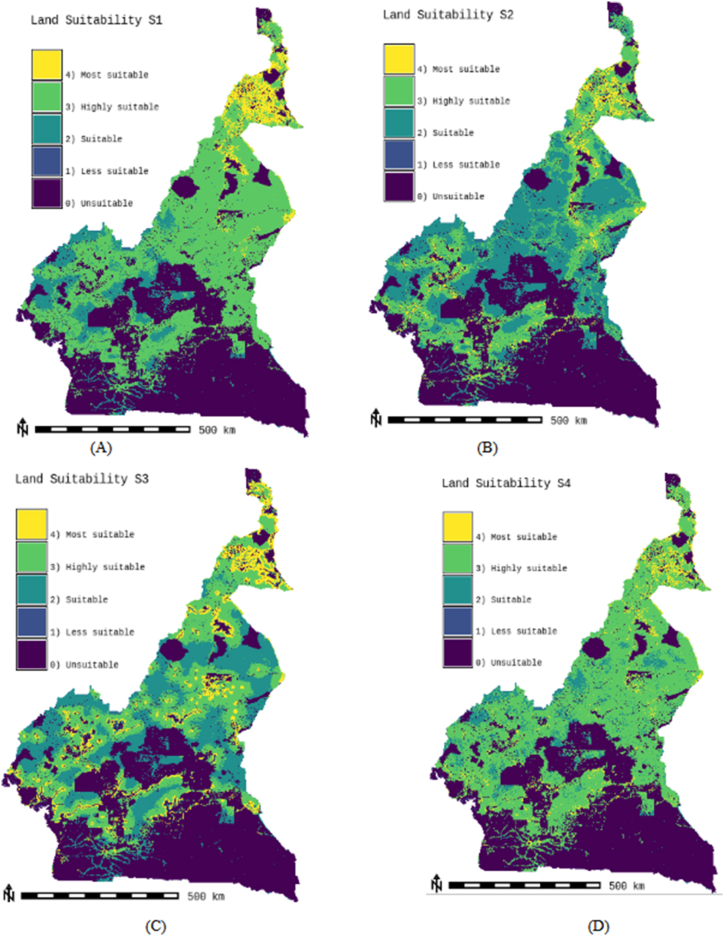
Land suitability scenario (A) Technical S1, (B) Economic S2, (C) Social S3 and (D) Equal-weight S4.

Sensitivity test reveals how each scenario affects the distribution of areas into various suitability categories. The categories include "unsuitable," "less suitable," "suitable," "highly suitable," and "most suitable." The total area of Cameroon remains constant across scenarios, but the proportion of land allocated to each suitability category changes depending on the weight emphasis. The unsuitable area remains constant across all scenarios at 42.40 % of the total land area. Scenario S2 (economic-focused) yields the highest percentage of land categorized as "suitable", at 29.34 %, compared to 13.66 % for S0. Scenario S1 (technical-focused) and S4 (equally weighted) exhibit similar percentages for highly suitable areas, around 43.53 % and 48.14 %, respectively, compared to 40.28 % for S0. Scenario S3 (social-focused) has the highest percentage of land in the most suitable category (7.13 %), indicating that social factors prioritize land that is more suitable for solar hydrogen production. S2 (economic) and S3 (social) deviate significantly from the base scenario (S0), especially in the "suitable" and "highly suitable" categories. For example, S2 shows 29.34 % of the land as "suitable," compared to only 13.66 % in S0. Similarly, S3 exhibits 23.25 % in this category. The technical scenario (S1) and equally weighted scenario (S4) show less variability compared to S0, particularly in the highly suitable class, with S1 at 43.53 % and S4 at 48.14 %, compared to 40.28 % for S0. The sensitivity analysis maps in Fig. 12 (A-D) demonstrate how focusing on different criteria (technical, economic, or social) significantly influences the classification of land for solar hydrogen production. The technical scenario (S1) map, shown in Fig. 12-A, exhibits more stability compared to the economic (S2) and social (S3) maps, while the equally weighted scenario (S4) reflects a balanced distribution. The sensitivity analysis reveals that the model is most sensitive to changes in economic and social criteria, as evidenced by significant shifts in land suitability in scenarios S2 and S3. In contrast, the technical criteria scenario (S1) demonstrates the most consistency with the baseline scenario (S0), suggesting that technical factors provide a more stable foundation for large-scale solar hydrogen site selection. These findings provide valuable insights for policymakers, indicating that emphasis on technical factors leads to more robust and consistent outcomes, whereas focusing on economic or social criteria introduces greater variability in land suitability assessments. Fig. 14 present graphical interpretation for land suitability areas for scenario test for hybrid solar-wind plants.

**Fig. 13:**
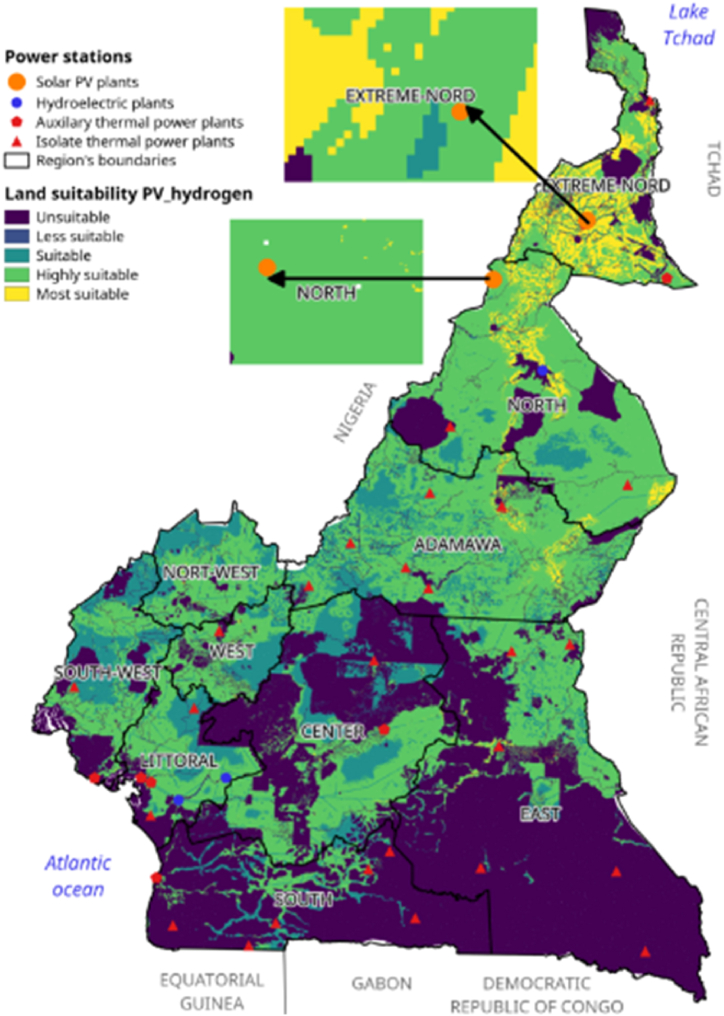
Validation map.

**Fig. 14:**
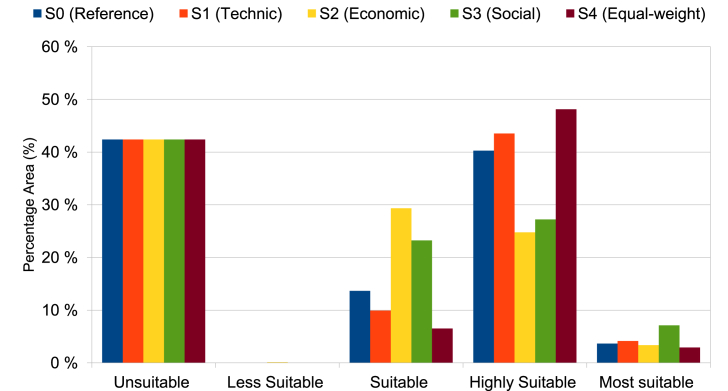
Graphical interpretation for land suitability areas for scenario test.

## 4 Conclusion

This study presents an in-depth analysis of three decision-making methodologies—AHP (Analytic Hierarchy Process), FAHP (Fuzzy Analytic Hierarchy Process), and MC-FAHP (Monte Carlo FAHP)—to evaluate optimal locations for solar hydrogen production in Cameroon. The comparative evaluation of these methodologies reveals that while all three provide similar overall estimates of energy potential, they differ significantly in their spatial distribution of suitable areas for solar and hydrogen production. Notably, FAHP and MC-FAHP identify a larger number of areas as "Highly Suitable" and "Suitable" for production, suggesting that these fuzzy approaches are more optimistic in their assessments. This highlights the strengths of fuzzy methods in managing uncertainties in the evaluation of criteria, compared to the more rigid, traditional AHP approach. The robustness of FAHP and MC-FAHP in accommodating uncertainty could prove to be an essential advantage as Cameroon navigates its energy transition.

The validation of solar power plants in Guider and Maroua—areas identified as "Highly Suitable" by the model—further corroborates the effectiveness and reliability of the proposed methodologies. These locations were confirmed to be ideal for solar energy development, reinforcing the ability of the models to accurately pinpoint regions with high solar energy potential. This study thus demonstrates that solar and hydrogen energy can play a pivotal role in addressing Cameroon's pressing energy needs, while contributing to economic development and environmental sustainability. Given the country's vast solar potential, solar hydrogen could serve as a key component of a diversified and sustainable energy strategy.

However, the study also identifies several challenges that could hinder the development and implementation of solar hydrogen energy projects. One of the primary obstacles is the availability and quality of data. The accuracy of the results is highly dependent on up-to-date spatial and thematic data, which is not always readily available. Additionally, the high initial investment costs and regulatory challenges could impede the large-scale deployment of these technologies. Therefore, the study underscores the need for targeted policies, strategic investments, and continued research and development efforts to overcome these barriers and fully harness the potential of solar and hydrogen energy.

A key insight from the sensitivity analysis is that the robustness of the proposed model varies depending on the emphasis placed on economic and social criteria. While technical factors, such as land suitability and solar potential, contribute to the stability of the model, the inclusion of economic and social factors introduces variability in the suitability assessments. This finding suggests that while the technical aspects provide a strong foundation for decision-making, economic and social considerations can offer additional layers of insight, which could be crucial for policymakers in aligning the energy development strategy with national priorities.

The study also highlights several limitations. The reliance on expert input for criterion weighting introduces an element of subjectivity, which could affect the robustness of the results. Future research should address this limitation by exploring alternative, more objective methods for determining criterion weights. Furthermore, the accuracy of the models could be enhanced by incorporating more advanced data sources, such as real-time satellite imagery and remote sensing technologies. The integration of these data sources could help to refine the spatial analysis and provide more accurate, up-to-date assessments of land suitability.

Moreover, while MC-FAHP is effective in managing uncertainty, further advancements in stochastic modeling and uncertainty quantification could improve decision-making accuracy. Future studies could focus on refining the economic and social criteria, which were found to be highly sensitive in this study. A deeper understanding of regional variations and specific regulatory challenges could improve the accuracy and reliability of the results. Additionally, further research could explore how policy frameworks and economic incentives could support the widespread adoption of solar hydrogen technologies, ensuring that the transition to renewable energy is not only technologically feasible but also economically viable.

In summary, this research provides a solid foundation for the integration of solar power and hydrogen production in Cameroon. The study offers a comprehensive and effective approach to identifying optimal sites for these energy technologies, contributing to the development of a sustainable and resilient energy infrastructure. By addressing the challenges identified in this study and implementing the recommendations provided, Cameroon can navigate the complexities of its energy transition and harness the potential of solar energy for the benefit of its economy and society. The adoption of these findings and policies will be crucial in achieving Cameroon's long-term energy sustainability goals.

## Declaration of Competing Interest

The authors declare that they have no known competing financial interests or personal relationships that could have appeared to influence the work reported in this paper.
